# Quality-Gated CycleGAN with Frequency-Split Composition for Structure-Constrained Contrast-Like Enhancement of Non-DICOM Chest CT: External Feasibility and Implications for Pulmonary Embolism AI

**DOI:** 10.3390/bioengineering13101145

**Published:** 2026-09-30

**Authors:** Sang-Hyeon Gill, Hyun-Young Choi, Yuseop Kim

**Affiliations:** 1 Department of Computer Engineering, Hallym University, Chuncheon 24252, Republic of Korea; rlftkdgus123@gmail.com; 2 Department of Emergency Medicine, Hallym University Gangnam Sacred Heart Hospital, Seoul 07441, Republic of Korea; chy6049@naver.com

**Keywords:** pulmonary embolism, non-DICOM CT, JPEG, contrast-like enhancement, CycleGAN, quality control, medical image processing, artificial intelligence, external testing, downstream evaluation

## Abstract

CT pulmonary angiography (CTPA) is the reference test for pulmonary embolism (PE), whereas AI datasets often contain display-domain (non-DICOM) non-contrast CT (NCT). We present a three-stage pipeline: a representation-consistency gate calibrated on DICOM-verified images (Stage 1), unpaired CycleGAN translation (Stage 2), and a frequency-split composition keeping the acquired finest spatial band plus support-guided residual refinement (Stage 3). Of 208 patients, 30 were held out. The final output reduced mediastinal distribution distance (14.92 to 7.19) and contrast-gap error (11.00 to 4.25; Holm-adjusted *p* < 0.001). Distribution metrics did not differ significantly from CUT, whereas three edge-based metrics favored the proposed method in all 30 patients. On 24 external NCT scans from three manufacturers (full-pipeline feasibility analysis without paired CTPA), distribution distances decreased and edge metrics again favored the method. The gate flagged all degraded external CTPA images (blur, down-sampling, heavy compression) in every calibration resample. An external 3D CTPA-trained PE model labeled unopacified arteries as embolus on NCT; enhancement reduced this volume in normal patients (12.0 to 1.7 mL), but detection stayed at chance, as on the original NCT (AUROC 0.47; original 0.42; CTPA 0.85). The pipeline harmonizes display-domain NCT with limited structural alteration for dataset preparation rather than CTPA-based detection.

## 1. Introduction

Pulmonary embolism is an acute thromboembolic disease in which a thrombus formed in the peripheral veins occludes the pulmonary arteries. Venous thromboembolism represents a substantial global disease burden of approximately ten million cases per year and is accompanied by considerable morbidity and mortality [1,2]. CT pulmonary angiography (CTPA) allows direct visualization of pulmonary arterial opacification and of intraluminal filling defects, and is therefore used as the reference standard for evaluating suspected pulmonary embolism [3]. CTPA, however, requires the administration of iodinated contrast media, and its use is limited in patients with impaired renal function or at risk of adverse reactions to contrast media [4,5]. Non-contrast CT (NCT) is an alternative in such cases, but its low intravascular contrast makes it difficult to delineate the pulmonary arterial lumen and the margins of a thrombus. AI-based image processing methods that support the analysis of pulmonary embolism imaging are therefore needed when the use of contrast media is restricted.

Deep learning studies on the automated detection of pulmonary embolism from CTPA have been pursued actively. PENet showed the feasibility of automated diagnosis of pulmonary embolism from volumetric CTPA [6], and a systematic review together with clinical evaluations of commercial AI algorithms has reported that deep learning models detect pulmonary embolism with a sensitivity comparable to that of radiologists [7,8,9]. The RSNA-STR public dataset has broadened this research ecosystem considerably [10]. Most of these approaches, however, presuppose contrast-enhanced CTPA, and are therefore not always directly applicable to practical data-construction settings in which the use of contrast media is restricted or in which registered paired NCT–CTPA data are scarce. In addition, a decrease in performance has been reported when a model trained on public data was applied to images from another institution [11], so that a procedure for handling the heterogeneity of input conditions is required before training.

The data format is a further practical constraint. DICOM is the standard format for quantitative CT analysis, but display-domain images such as JPEG and BMP are frequently distributed and used for reasons of public dataset construction, retrospective data sharing, de-identification, and accessibility. MIMIC-CXR-JPG, derived from the large-scale chest radiograph database MIMIC-CXR, is a representative example that provides JPG files together with structured labels [12,13]. In display-domain images, however, information on the true Hounsfield unit (HU) values, window/level settings, and bit depth is limited, and because pixel intensity in CT corresponds directly to HU, this loss of information may lead to a loss of diagnostic information [14,15,16]. Recent reporting guidelines for medical imaging AI likewise emphasize explicit reporting of preprocessing for reproducibility and transparency [17,18]. An assessment of the reliability of the input images should therefore precede the use of non-DICOM CT in AI synthesis models.

One approach explored when contrast media cannot be used is to synthesize contrast-like images from non-contrast CT. GAN-based models have been reported to generate synthetic contrast enhancement from non-contrast chest CT [19], but conditional translation such as pix2pix requires registered paired data [20]. In routine clinical practice, non-contrast CT and CTPA are acquired at different time points and in different positions, so that registered pairs are difficult to obtain. CycleGAN can learn domain translation without paired data through cycle-consistency and identity losses [21] and is therefore suited to unpaired translation, but it relies on global intensity and texture transformation and does not sufficiently correct peripheral vascular branches and small-vessel signal. Diffusion models, meanwhile, have attracted attention in medical image synthesis and restoration because of their strong generative priors [22,23]; applying generative correction to an entire slice, however, may also alter non-target structures outside the vessels and cardiac chambers. In the present study, the local refinement that follows the global translation was therefore designed as a low-resolution residual refinement rather than a generative model, guided by a learned soft gate that concentrates the correction on a vascular support, while the finest spatial band is inherited from the acquired image.

Taken together, these observations indicate that input screening and a structure-constrained local refinement need to be integrated into a single workflow in the non-DICOM, unpaired CT setting. To this end, we propose a quality-guided contrast-like enhancement framework consisting of three stages. First, a representation-consistency gate labels display-domain slices QC-pass or QC-flag. Its reference feature ranges are estimated from display images whose correspondence to the original DICOM was verified slice by slice, using intensity, entropy, edge, histogram and saturation features. The gate is intended to screen inputs whose representation departs from the calibrated display convention or that carry controlled degradations; it is not a measure of diagnostic image quality. Second, QC-pass non-contrast CT is translated into a global contrast-like domain by a CycleGAN trained in an unpaired manner. Third, a frequency-split composition keeps the finest spatial band of the acquired image in the output of the global translation, after which a low-resolution residual refinement guided by a learned soft gate corrects the local signal distribution around the vascular support.

The main contributions of this study are as follows. First, we constructed a representation-consistency gate for display-domain CT whose reference images were verified against the original DICOM, characterized what it does and does not measure, and tested its calibration stability and its behavior on external data. Second, we combined an unmodified CycleGAN with a frequency-split composition in which the finest spatial band is inherited from the acquired image, and quantified the trade-off between contrast-like intensity distribution and alteration of the input structure under a patient-level held-out protocol, in comparison with CLAHE and CUT and on external non-contrast CT. Third, we tested whether the enhanced 2D display images can take the place of CTPA as the input of an existing 3D PE detector trained on CTPA volumes, with controls for the change in format and along the detector’s full operating range. This defines the scope of the method for PE AI: the enhancement reduced the extent to which non-opacified arteries were labeled as embolus, whereas detection did not change, as on the original non-contrast DICOM, because the embolus–blood contrast is absent from non-contrast acquisitions.

This study presents a reproducible workflow for display-domain data in settings where original DICOM files or registered paired data are limited. Our aim is to characterize how far contrast-like representation harmonization can be taken with limited structural alteration, and what it provides for the construction of PE AI datasets from non-DICOM images.

## 2. Materials and Methods

### 2.1. Study Design and Overall Framework

This study was designed as a retrospective study to evaluate the feasibility of quality-controlled contrast-like image enhancement for display-domain chest CT images in a data setting where original DICOM files or spatially registered paired NCT–CTPA data are limited. The scope of this study is the conversion of the representation of display-domain images into a contrast-like representation; recovery of true HU values, generation of diagnostically equivalent CTPA, and replacement of contrast-enhanced CT examinations are not objectives of this study.

**Figure 1:**
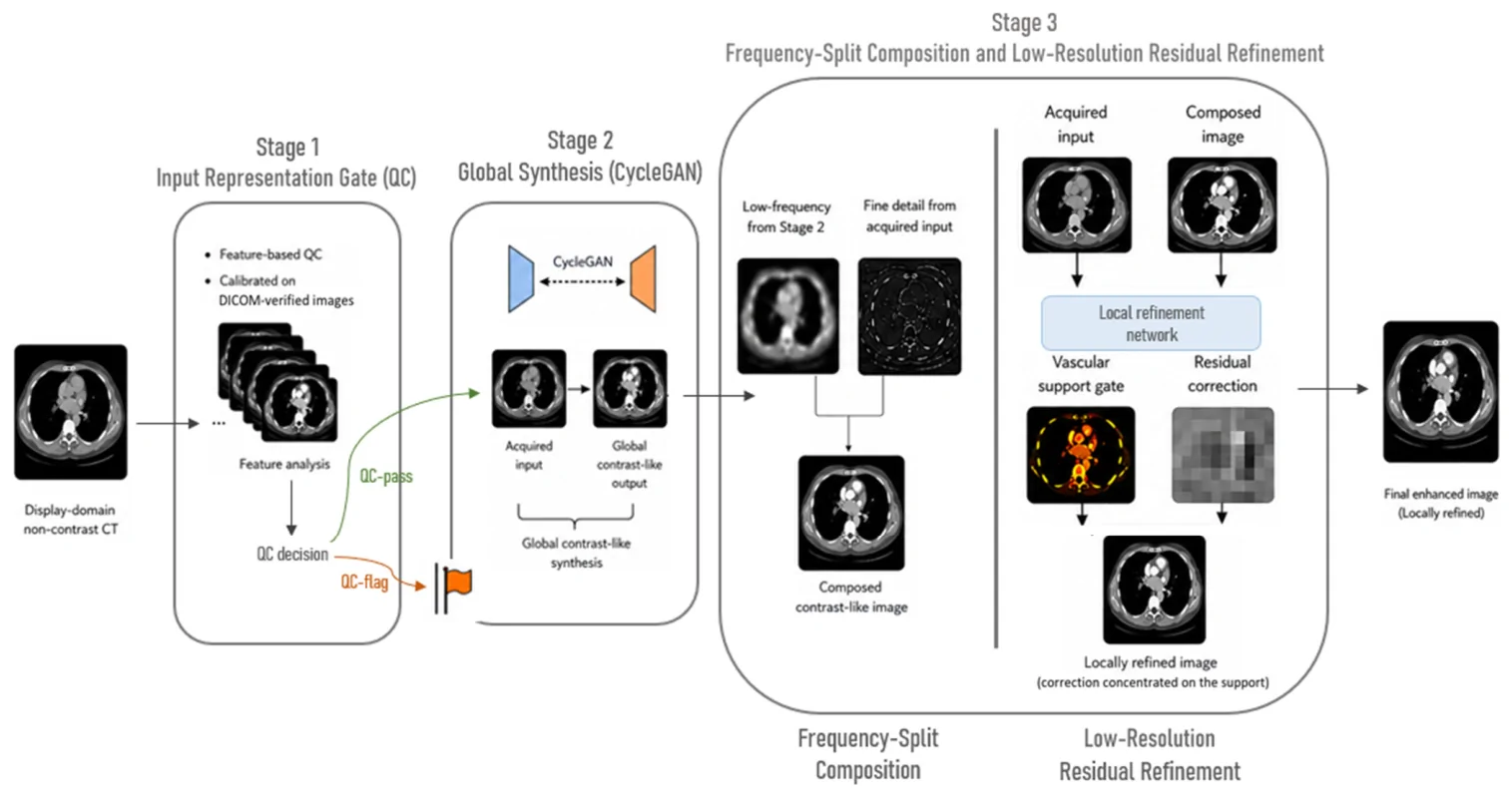
Overall framework of the proposed three-stage contrast-like enhancement pipeline. A display-domain non-contrast CT slice first passes the Stage 1 representation-consistency gate calibrated on DICOM-verified display images and is labeled QC-pass or QC-flag. QC-pass slices are translated into a global contrast-like image by an unmodified CycleGAN generator (Stage 2). Stage 3 post-processes the frozen Stage 2 output in two components: frequency-split composition recombines it with the acquired input so that the finest spatial band is inherited from the acquired image, and a low-resolution residual refinement guided by a learned soft gate then corrects the local signal distribution, with the correction concentrated on the vascular support.

The proposed framework consists of three stages integrated into a single pipeline (Figure 1). Stage 1 is a representation-consistency gate that separates display-domain inputs into QC-pass and QC-flag on the basis of image features calibrated on display images verified against the original DICOM. Stage 2 translates QC-pass non-contrast CT into a global contrast-like domain using a CycleGAN trained in an unpaired manner. Stage 3 post-processes the frozen Stage 2 output in two components: a frequency-split composition keeps the finest spatial band of the acquired image, after which a low-resolution residual refinement guided by a learned soft gate corrects the local signal distribution around the vascular support.

The input gate was used to compose the training inputs and to determine input eligibility at deployment. The training, validation, test and lesion cohorts were split at the patient level (split seed 20260817). The 30-patient test cohort was not used for model selection; it was used for the pre-specified final evaluation, and the sensitivity analyses requested during peer review (Section 3.3 and Section 3.6) were also evaluated on it without re-selecting any model, epoch or parameter.

This study was approved by the Institutional Review Board of Hallym University Kangnam Sacred Heart Hospital [IRB no. HKS 2025-05-010-003], and the requirement for informed consent was waived because the study involved retrospective, de-identified image analysis. All images were de-identified before analysis, and the external public dataset was used in accordance with its terms of use.

### 2.2. Dataset Construction and Study Cohorts

This study used a display-domain chest CT cohort from our institution, split at the patient level. The images are grayscale PNG files converted from the original JPEG/BMP images with pixel values preserved, comprising 365 studies, 208 patients, and 93,133 slices (Table 1). The split was performed by patient identifier so that multiple studies from the same patient were not assigned to different splits. Eleven patients whose pulmonary embolism lesions had been annotated by a radiologist were excluded from training and testing and formed a lesion cohort. Annotation folders were available for 20 study folders, which corresponded to 18 unique examinations (two examinations had been annotated twice in separate folders); the non-contrast annotations comprised 248 lesion boxes on 176 annotated images. The lesion cohort had been inspected during the design of the composition step (Section 2.6.1) and is therefore reported as an exploratory cohort, not as an independent confirmation set. Thirty patients formed the test cohort described above.

For Stage 1, display images whose original DICOM series were available were used as the reference (calibration) subset: 32 normal non-contrast series from 25 patients who were not part of the 208-patient cohort, and 18 contrast and 18 non-contrast series from the 11 lesion-cohort patients. Display files share the file names of the DICOM instances, which allowed exact slice correspondence (Section 2.4.1). For a calibration sensitivity analysis, 109 further DICOM-verified series (55 contrast, 54 non-contrast) from 30 training/validation patients were added (Section 2.4.3); test and lesion patients were never used for calibration. External data: (i) 76 RSNA-STR CTPA studies (1824 slices; 24 evenly spaced slices from the central 15–85% of each study) [10], used to test Stage 1 and as an external contrast-enhanced reference; (ii) 24 non-contrast chest CT series from the LIDC-IDRI collection [24,25] (8 GE [GE HealthCare, Chicago, IL, USA], 8 Siemens [Siemens Healthineers, Erlangen, Germany], 8 Toshiba [Toshiba Medical Systems, Otawara, Japan]; selected by a rule fixed before download, Section 2.7), used for an external feasibility analysis of the complete pipeline; and (iii) the 25 normal patients above (32 non-contrast and 32 CTPA examinations), used as the PE-negative group of the downstream analysis.

### 2.3. Image Preprocessing and Format Harmonization

DICOM images were converted to Hounsfield units according to Equation (1).

HU = pixel × RescaleSlope + RescaleIntercept(1)

From the reconstructed HU images, three windows were generated following clinical convention: a lung window (center −600, width 1500), a mediastinal window (center 40, width 400), and a vascular/PE window (center 100, width 700). Each windowed image was linearly normalized to an 8-bit image in the range [0, 255]. This windowed reference is used in Stage 1 as the representation that a faithfully converted display image is expected to have. Slice-by-slice comparison with the DICOM showed that the institutional display images are window renderings of the DICOM followed by lossy JPEG encoding: for each series, a grid search over window width (300–1600 HU) and level (−100 to 300 HU) identified the display window, which reproduced the display images with a median absolute error of 0.17 gray levels for the normal series (lossless BMP export) and 1.77–1.79 gray levels for the PE series (JPEG export). The identified windows were width 400/level 25 HU for most PE series and width 525–530/level 70 HU or 480/50 HU for the normal series; the PE exports were reproduced to 0.43 gray levels by this window followed by JPEG encoding at quality 75.

Non-DICOM images (JPEG/BMP) were loaded as single-channel grayscale; those stored as RGB were converted to a single channel through a luminance transform. All images were resized to 512 × 512 and unified to 8-bit, and intensities were normalized to [0, 1]. Because the entire institutional cohort was provided as display-domain images without DICOM, only the same grayscale conversion, resizing, and normalization were applied throughout training and evaluation. All preprocessing parameters are summarized in Table 2; the preprocessing steps, including normalization, resizing, bit-depth change, and window/level adjustment, are reported following the CLAIM 2024 guideline [17].

### 2.4. Stage 1: Representation-Consistency Gate Calibrated on DICOM-Verified Display Images

**Figure 2:**
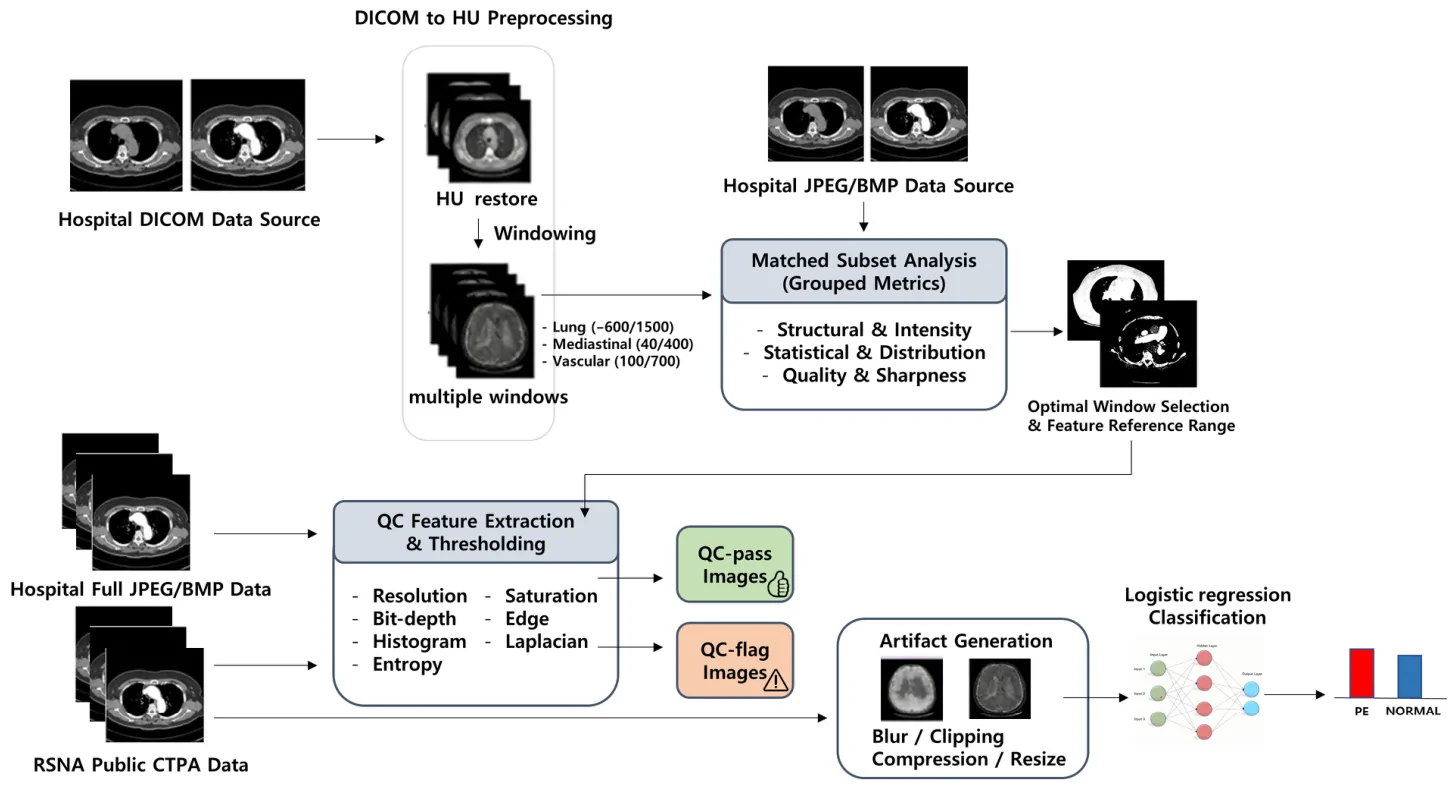
Representation-consistency gate for non-DICOM CT (Stage 1). (Section 2.4.1) DICOM images from the matched subset are converted to Hounsfield units and rendered into three window references (lung, mediastinal, vascular). Display slices are matched to the DICOM instance with the same file name (order verified against the image-position coordinate), the best window is selected by the highest mean SSIM, and a reference histogram template is derived. (Section 2.4.2) Six image features—entropy, extreme-value (0/255) fraction, mean Sobel edge magnitude, Laplacian variance, and histogram distance/correlation to a template—are extracted from the non-DICOM cohorts and compared with one-sided thresholds (2.5th percentile for lower-bound rules, 97.5th percentile for upper-bound rules) estimated from the DICOM-verified reference images to label QC-pass and QC-flag slices and to compute a case-level flag ratio. For DICOM-absent cohorts, only intrinsic features and the reference histogram template are applied.

The overall procedure of Stage 1 is presented in Figure 2. The representation-consistency gate derives its reference statistics from display images verified against the original DICOM (Section 2.4.1) and applies one-sided thresholds defined on this basis to non-DICOM images in order to obtain a slice-level QC-pass/QC-flag label and a case-level flag ratio (Section 2.4.2).

In this study, the gate was applied to all 93,133 slices, and only QC-pass slices were used to compose the training data.

#### 2.4.1. DICOM Correspondence and Display-Window Identification

The display files of each series carry the file names of the DICOM instances, and their order runs from superior to inferior. Each display slice was therefore compared with the DICOM instance of the same name, and the correspondence was verified by the image-position coordinate. Each display slice was compared with the DICOM rendered in three conventional windows (lung, mediastinal and vascular; Section 2.3) and in the window identified for that series (Section 2.3) using SSIM [26], PSNR, histogram distance and entropy difference. The conventional window with the highest mean SSIM was recorded as the best-matching window of the series. The DICOM comparison serves to verify that the reference display images are faithful renderings; the gate itself operates only on display-image features.

#### 2.4.2. Gate Features and Decision Rule

Four intrinsic features and two template features were computed for every display slice: Shannon entropy; the fraction of pixels at the extreme gray levels 0 or 255 (which includes background air and therefore also reflects field of view and body coverage); mean Sobel edge magnitude; Laplacian variance [27]; and the Wasserstein distance and correlation between the slice histogram and a template, defined as the mean histogram of the reference display images of the same group and phase. A slice was flagged when any of six one-sided rules was met: entropy, Sobel edge magnitude, Laplacian variance or histogram correlation below the 2.5th percentile of the reference distribution, or extreme-value fraction or histogram distance above the 97.5th percentile. Because each rule excludes 2.5% of the reference images by construction, the gate flags 8.6–10.8% of its own reference images (in-sample pass rates 89.2%, 91.2% and 91.4% for the normal non-contrast, PE contrast and PE non-contrast profiles). Institutional non-contrast and contrast slices were assessed with the PE non-contrast and PE contrast profiles, respectively, and the proportion of flagged slices was computed for each case.

The gate therefore addresses two properties: consistency of the input representation with the calibrated display convention, and response to controlled degradation (Section 3.1.4). Neither is a measure of diagnostic image quality, for which no clinical reference was available.

#### 2.4.3. Calibration Stability Analyses

Because the reference subset is small, calibration uncertainty was assessed in three ways, none of which changed the deployed thresholds: (i) 1000 patient-block bootstrap replicates of the PE contrast profile (template and six thresholds re-estimated in every replicate, repeated studies kept within patients), applied to the unmodified and degraded external images; (ii) leave-one-patient-out re-estimation; and (iii) re-estimation after adding the 109 DICOM-verified training/validation series (41 patients in total). The dependence of the external pass rate on the display conversion was assessed by rendering the same external DICOM slices with the submitted conversion (width 700/level 100 HU) and with the institutional export convention (width 400/level 25 HU, with and without JPEG quality 75).

### 2.5. Stage 2: CycleGAN-Based Global Contrast-Like Enhancement

CycleGAN [21] was used for unpaired translation between QC-pass non-contrast CT (domain X) and contrast CT (domain Y). The model comprises two generators, G: X→Y and F: Y→X, and two discriminators, D_X_ and D_Y_. Each generator has a ResNet-9blocks [28] architecture that limits information loss through residual connections, and each discriminator is a 70 × 70 PatchGAN that distinguishes real from fake.

Training was performed with a combination of three loss functions. First, least-squares adversarial losses were used [21]; the generator minimizes E_x_[(D_Y_(G(x)) − 1)^2^] and the discriminator minimizes E_y_[(D_Y_(y) − 1)^2^] + E_x_[D_Y_(G(x))^2^] (Equation (2)); the same form applies to F and D_X_.

L_GAN_(G,D_Y_) = E_y_[(D_Y_(y) − 1)^2^] + E_x_[D_Y_(G(x))^2^] (2)

Second, the cycle-consistency loss constrains the images reconstructed through both generators to agree with the originals (Equation (3)).

L_cyc_ = E_x_[‖F(G(x)) − x‖_1_] + E_y_[‖G(F(y)) − y‖_1_] (3)

Third, the identity loss suppresses unnecessary intensity changes and structural distortion within the input domain (Equation (4)).

L_id_ = E_y_[‖G(y) − y‖_1_] + E_x_[‖F(x) − x‖_1_] (4)

The final objective is given in Equation (5), with λ_cyc_ = 10 and λ_id_ = 5 (identity weight 0.5 relative to λ_cyc_, as in the official implementation).

L = L_GAN_ + λ_cyc_L_cyc_ + λ_id_L_id_ (5)

The cycle-consistency and identity losses encourage the transformation of the global contrast tone while preserving the anatomical structure of the vessels and the surrounding tissue without paired labels.

Epoch selection was performed on the validation cohort only. The primary metric was the patient-level median intensity distribution distance; among the epochs satisfying both the structure gate (EPI ≥ 0.55, ROI sharpness ratio 0.8–1.6) and the validity gate (distribution distance lower than that of the input, and contrast gap ≥ 12), the epoch with the lowest value of the primary metric was selected. The non-contrast-to-contrast-like output of CycleGAN (x_stage2_) becomes the input for Stage 3. Generator inputs were scaled to [−1, 1].

### 2.6. Stage 3: Frequency-Split Composition and Low-Resolution Residual Refinement

**Figure 3:**
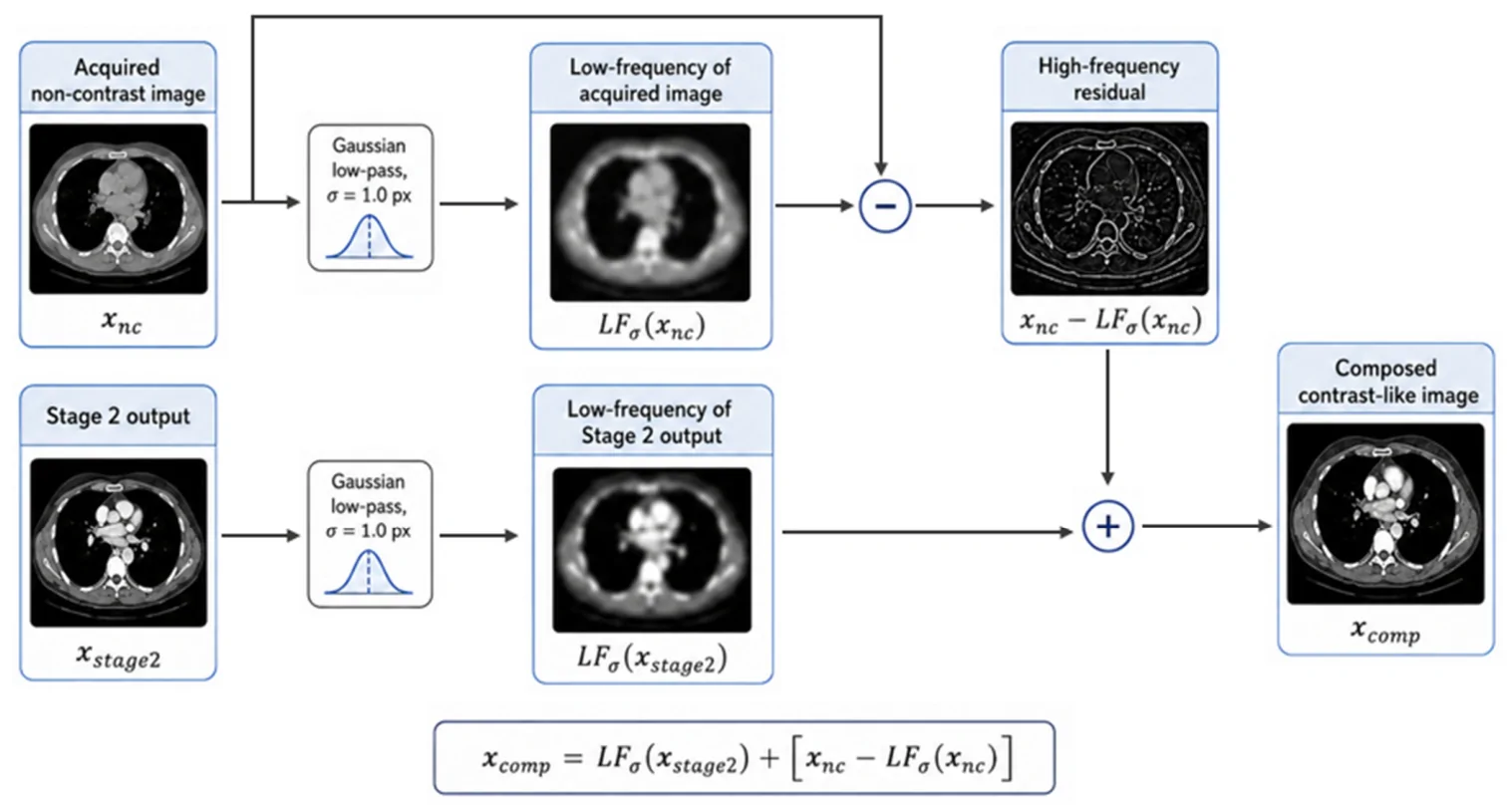
Frequency-split composition (Stage 3, first component). The Stage 2 output is low-pass-filtered with a Gaussian kernel (σ = 1.0 px) and recombined with the high-frequency residual of the acquired non-contrast image. The acquired image is modified only by the Gaussian-smoothed difference to the Stage 2 output, so the finest spatial band is inherited from the acquired image while coarser, contrast-like changes are transferred.

**Figure 4:**
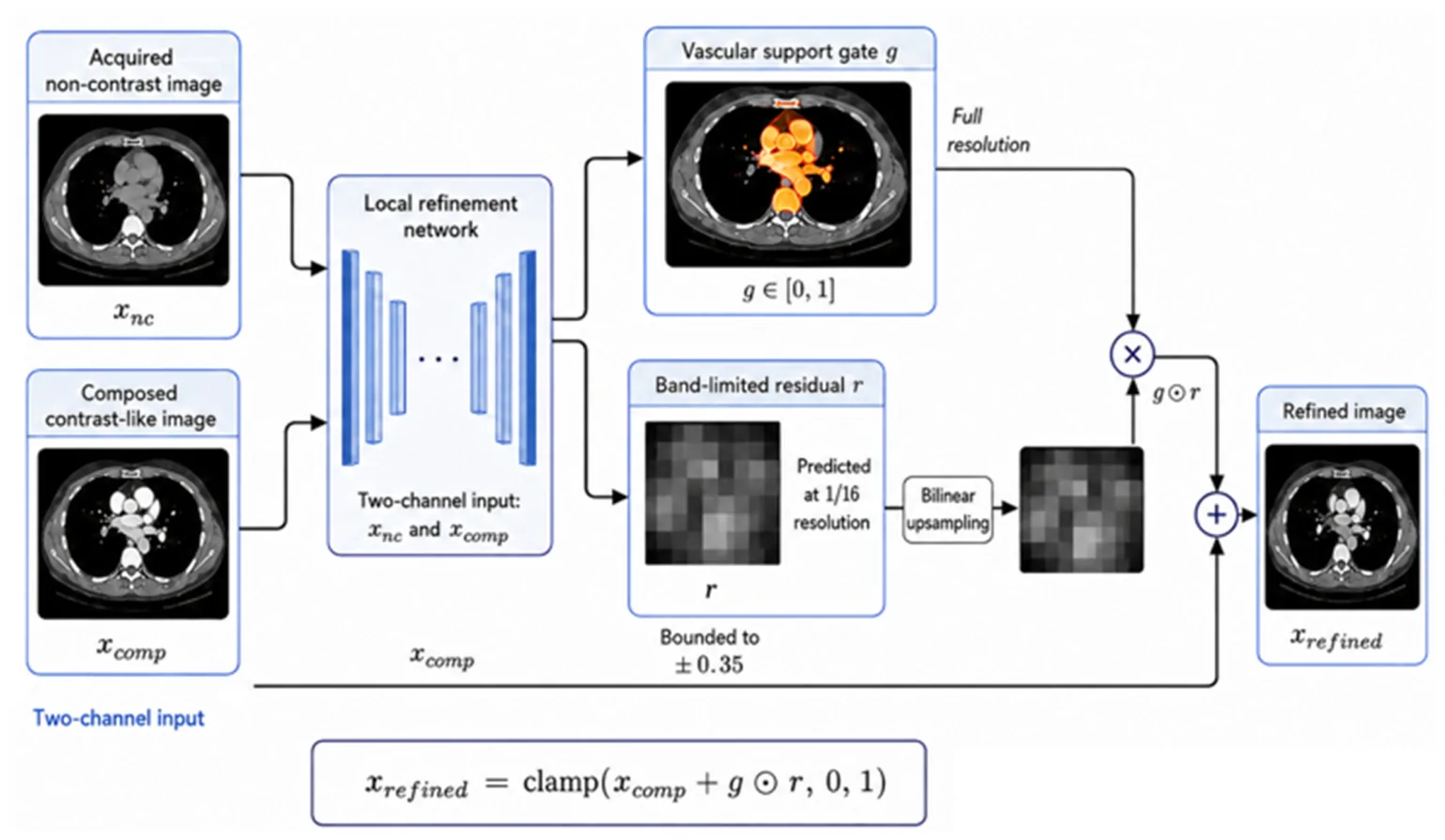
Low-resolution residual refinement (Stage 3, second component). The composed image and the acquired non-contrast image form a two-channel input. The network predicts a vascular support gate at full resolution and a residual bounded to ±0.35 at 1/16 resolution, which is bilinearly up-sampled; the refined image is obtained by adding the gated residual to the composed image. The residual head is zero-initialized so that training starts from the identity mapping; the gate is learned without a segmentation label. The orange overlay in the vascular support gate panel shows the learned gate g superimposed on the non-contrast image.

Stage 3 post-processes the frozen Stage 2 output in two components. First, frequency-split composition inherits the finest spatial band from the acquired image (Section 2.6.1; Figure 3); then, a low-resolution residual refinement guided by a learned soft gate corrects the local signal distribution around the vascular support (Section 2.6.2; Figure 4).

#### 2.6.1. Frequency-Split Composition

It is known that a generator using transposed convolutions can create fine-band components during up-sampling when the kernel size is not divisible by the stride [29,30]. In the generator used here, the residual blocks likewise operate at one-quarter of the input resolution, so that the fine band of the Stage 2 output originates entirely from the two transposed convolutions of the decoder (Section 3.3).

Using the fact that the contrast effect exists in the coarse spatial frequencies as an intensity shift over the vessels and the cardiac chambers, the Stage 2 output x_stage2_ was recombined with the acquired non-contrast image x_nc_ as follows.

x_comp_ = LF_σ_(x_stage2_) + [x_nc_ − LF_σ_(x_nc_)], σ = 1.0 px (6)

Equation (6) can be written equivalently as

x_comp_ = x_nc_ + LF_σ_(x_stage2_ − x_nc_) (7)

i.e., the acquired image is modified only by a Gaussian-smoothed difference. The Gaussian transfer function exp(−2π^2^σ^2^f^2^) passes 0.72% of the generator–input difference at the Nyquist frequency (2-pixel period), 29% at a 4-pixel period and 74% at an 8-pixel period for σ = 1 px. Components of the difference at the finest scale are therefore strongly attenuated rather than removed by an ideal cut-off, and coarser structure is determined by the generator. Of the widths examined (0.5–2 px), σ = 1 px is the smallest that attenuates the Nyquist-band difference, the band in which the transposed-convolution decoder produces its fine-band components (Section 3.3), by more than 99% (σ = 0.75 px passes 6.2% and σ = 0.5 px 29%). σ was fixed before the test cohort was analyzed and was not re-selected after the sensitivity analysis (Section 3.3). Clipping to the 8-bit display range introduces a deviation of 0.18 gray levels on average in 1.29% of the mediastinal ROI pixels.

#### 2.6.2. Support-Guided Low-Resolution Residual Refinement

The composed image is then refined locally by a four-level U-Net (width 48; 4.33 × 10^6^ parameters) that receives two channels, x_nc_ and x_comp_, and outputs

x_refined_ = clamp(x_comp_ + g ⊙ r, 0, 1) (8)

where g ∈ [0, 1] is a full-resolution sigmoid gate and r is a residual bounded to ±0.35 that is predicted at 1/16 resolution and bilinearly up-sampled. The residual head is zero-initialized so that training starts from the identity mapping (the gate is initialized at 0.5). No vessel segmentation is performed at inference: the gate is learned end to end, and the refiner requires only the non-contrast image and the composed image.

The vascular support is used only during training and evaluation. It is defined on each training slice as the pixels of the mediastinal region of interest (body mask of the non-contrast image intersected with a fixed central ellipse) whose intensity in the translation-registered contrast image of the same study is in the upper 20% within that region, dilated by a 5 × 5 structuring element. It is an intensity-based surrogate of the opacified vascular and cardiac lumen rather than a validated pulmonary-artery segmentation. The loss comprises a mean-matching term over 32 × 32 regions inside the support, a differentiable one-dimensional Wasserstein term on the mediastinal intensity distribution, a preservation term |x_refined_ − x_comp_| on the body outside the support dilated by 9 × 9 (weight 2), and a gate sparsity term outside the support (weight 0.05). The refiner was trained for up to 8 epochs, and the epoch was selected on the validation cohort (epoch 1). Its cost per 512 × 512 slice is 82.7 × 10^9^ multiply–accumulate operations in convolutions and 16.1 ms (median of 50 runs, batch size 1, NVIDIA A100 80 GB, excluding data transfer and the other stages), with 397 MiB peak GPU memory.

### 2.7. Experimental Protocol and Comparison Methods

(E1) Behavior of the Stage 1 gate: DICOM correspondence, calibration stability and external testing. (E2) Distribution matching and input-structure agreement of the final output on the test cohort. (E3) Effect of the frequency-split composition, including sensitivity to σ and multi-scale input–output agreement. (E4) Effect and spatial extent of the residual refinement. (E5) Local contrast within radiologist-annotated lesion boxes relative to intensity-matched control boxes. (E6) Use of the enhanced images as input to an independently developed, open-weight 3D PE model trained on CTPA (Section 2.8). (E7) External feasibility of the complete pipeline on public non-contrast CT: LIDC-IDRI series with 90–180 images, one per patient, taken in patient-ID order within vendor and accepted as non-contrast when the 90th percentile of mediastinal HU on mid-thoracic slices was below 100 HU and no contrast agent was recorded, up to 8 per vendor; the rule was fixed before download. After screening and before any processing, the contrast-agent condition was clarified so that a tag explicitly recording no IV contrast (‘W/O IV CONTRAST’) counted as no agent recorded; this added eight Toshiba series and changed no other decision. One Philips series fell in the candidate range and was excluded by the HU criterion. External series were rendered with the institutional export convention (width 400/level 25 HU, JPEG quality 75). (E8) Robustness: patient-level effect sizes, repeated training runs, adjacent-slice consistency and objectively selected failure cases. A standard display transform (CLAHE [31,32]) and an unpaired translation baseline (CUT [33]) were used as comparison methods.

### 2.8. Evaluation Metrics and Statistical Analysis

Intensity distribution matching was measured using the Wasserstein-1 distance (earth mover’s distance) of gray-level histograms over the mediastinal ROI (roi_emd) [34], the body (body_emd) and the vascular support (support_emd), each against the registered CTPA of the same patient. The contrast gap is the median gray level of the mediastinal ROI minus that of the chest-wall soft tissue outside the dilated mediastinal ellipse; gap_error is its absolute difference from the same patient’s CTPA. All masks are defined on the non-contrast input, so that they do not adapt to the output. Input-structure agreement was measured using the edge preservation index (EPI; Jaccard overlap of Canny edge maps of output and input), the ratio of Laplacian variance in the mediastinal ROI between output and input (sharpness), and the fractions of output edges absent from the input (novel edge) and of input edges absent from the output (lost edge), with a 5 × 5 tolerance. These are edge-based proxies and not anatomical ground truth. FID [35] was reported as a cohort-level metric. For external NCT, distribution metrics were computed against the pooled RSNA-STR CTPA reference rendered with the same conversion, because no paired CTPA exists.

All comparisons were made at the patient level: slice values were aggregated within each patient (histograms pooled; EPI median; edge rates mean), and each patient contributed one value. We report the median paired difference with a 95% percentile bootstrap confidence interval over patients (20,000 resamples) and the paired Wilcoxon signed-rank test [36] with Holm correction [37] within each metric family. Study-level AUROC was computed with a patient-cluster bootstrap (5000 resamples, stratified by group).

#### Evaluation with an Independent CTPA-Trained PE Model

This analysis asks whether the enhanced images can take the place of CTPA as the input of an existing PE detector. The detector is a 3D model trained on CTPA volumes in Hounsfield units, whereas the pipeline processes 8-bit display images slice by slice; the test therefore also involves a change in format, which was controlled as described below. We used a publicly released 3D residual-encoder U-Net for PE segmentation trained with nnU-Net v2 on 430 CTPA patients from another institution (Hugging Face yzluka/PulmonaryEmbolismSegmentation, commit 7ce235f; CC BY-NC 4.0) [38], without fine-tuning and with its published preprocessing (HU clipping [−195, 305], resampling to 1.0 × 0.74 × 0.74 mm, sliding-window inference). PE-positive examinations were the 18 unique examinations of the lesion cohort (11 patients, not used to train Stage 2 or 3; Section 2.2); PE-negative examinations were the 32 examinations of the 25 normal patients, who had contributed to the Stage 1 calibration only and were not used to train or select Stage 2 or 3. Volumes were ordered by the DICOM image position. Every display-derived input (raw non-contrast, CLAHE, CUT, Stage 2 with composition, final output, and real CTPA) was mapped to HU by the same inverse window (width 400/level 25). Because the normal exports used a different window and lossless format, the normal non-contrast series were rendered from their DICOM with the PE export convention (width 400/level 25, JPEG quality 75), so that PE and normal inputs share window and encoding. The complete volumes were enhanced slice by slice and stacked in DICOM order. Two references separate the change in format from the image content: real CTPA rendered to display images and passed through the same conversion, and the original non-contrast DICOM used without any conversion; real CTPA on the original DICOM served as the reference for the model itself. The primary end point was study-level discrimination (AUROC of the predicted PE volume, *p* > 0.5); secondary end points were the predicted PE volume in normal examinations (false-positive burden) and the fraction of radiologist lesion boxes intersected by the predicted PE mask on the same slice (lesion-box sensitivity). The end points were fixed in advance and changed once, before any enhanced input was processed: a sanity run on two true-HU examinations showed that the model labels non-opacified vessels as PE, so the primary end point was moved from lesion-box sensitivity to study-level AUROC (Supplementary Materials). Two display CTPA series whose display–DICOM correlation was below 0.9 (a different reconstruction) were excluded from the display-CTPA reference only. Because sensitivity and false-positive burden cannot be separated at a single threshold, the stored probability maps were also re-thresholded over the full range (8-bit thresholds 1–254), and the representations were compared at matched false-positive burdens of 0.5, 1, 2, 5 and 10 mL per normal examination, with each threshold chosen on the normal group alone; this analysis was specified in the protocol before any threshold other than 0.5 was applied. False-positive candidates were counted as connected components of at least 100 mm^3^ in normal examinations. As a replication, the analysis was repeated on 28 further PE examinations with DICOM of both phases from 16 patients not used to train Stage 2 or 3 (3 lesion-cohort, 7 validation and 4 test patients, and 2 patients outside the development cohort; 24 of the 28 examinations were not part of the primary analysis) against the same normal group. These examinations had no lesion annotations, so only study-level discrimination was assessed; examinations without an institutional export were rendered from DICOM with the export convention, and, because follow-up examinations may no longer contain emboli, the analysis was also restricted to the earliest examination of each patient.

### 2.9. Implementation Details

All models were trained on an NVIDIA A100 GPU (NVIDIA Corporation, Santa Clara, CA, USA) under Python 3.9, PyTorch 2.8, and CUDA 12.8. CycleGAN was trained with the Adam optimizer [39] (learning rate 0.0002, β_1_ 0.5), a batch size of 4, ngf 64, ndf 64, and an input size of 512 × 512, for a total of 30 epochs (15 epochs of training followed by 15 epochs of linear decay). The final checkpoint was fixed at epoch 13, selected on the validation cohort according to the rule described in Section 2.5. The normal ranges of the Stage 1 gate features were the one-sided 2.5th/97.5th percentiles of the DICOM-verified reference images and applied to all display-domain images. The data split was performed at the patient level (split seed 20260817). The seed of the submitted CycleGAN run was not recorded; four additional runs with seeds 101, 202, 303 and 404 were trained for robustness (Section 3.6).

## 3. Results

### 3.1. Representation-Consistency Gate (Stage 1)

#### 3.1.1. Window Correspondence of Non-DICOM CT Images

On the DICOM-verified subset, each display slice was compared with the same DICOM instance rendered in three conventional windows and in the window identified for its series (Table 3). The identified windows reproduced the display images with median absolute errors of 0.17 (normal) and 1.77–1.79 gray levels (PE; lossy JPEG export), showing that the institutional display images are faithful window renderings of the DICOM. Among the conventional windows, the mediastinal window was the closest for 16/18 PE contrast and 15/18 PE non-contrast series, and the vascular window for 28/32 normal series, consistent with the identified windows (PE mostly width 400/level 25; normal width 525–530/level 70). The unknown element in display-domain data is therefore which window was used, and this differed between export batches of the same institution.

#### 3.1.2. QC Outcomes Across the Non-DICOM Cohort

Applying the one-sided thresholds derived from the DICOM-verified reference images (Section 2.4.2) to all 93,133 slices of the institutional cohort, the overall QC-pass rate was 0.894 (Table 4). By phase, it was 0.892 for non-contrast and 0.896 for contrast. In both phases, the main flag causes were a high extreme-value fraction (7.9% for non-contrast, 7.6% for contrast) and low entropy (6.5% for non-contrast, 7.3% for contrast); flags were concentrated in a minority of series (median per-series pass rate 1.00; 47 of 729 series below 0.50). The designed in-sample flag rate of the reference images was 8.6–10.8% (Section 2.4.2). The splits are the same as in Table 1.

#### 3.1.3. QC Validity on the Matched Subset

To test whether the gate separates display images by their agreement with the DICOM, QC-pass and QC-flag slices of the DICOM-verified subset were compared against the same DICOM instance rendered in the identified window, with a patient-level paired analysis restricted to patients having both labels (Table 5). No metric differed significantly between QC-pass and QC-flag slices after Holm correction (all adjusted *p* ≥ 0.14). Because the institutional display images are faithful renderings (Section 3.1.1), the gate’s flags in this subset reflect atypical feature values relative to the reference distribution—such as a high extreme-value fraction or low entropy—rather than a departure from the DICOM rendering. The gate’s response to a changed representation or to degradation was therefore tested with controlled perturbations and alternative conversions (Section 3.1.4).

#### 3.1.4. External Artifact Sensitivity

The gate was tested on external CTPA that we converted to the display domain (RSNA-STR, 76 studies, 1824 slices), with thresholds unchanged. With the submitted conversion (width 700/level 100), the pass rate was 0.884 for PE studies, 0.798 for PE-negative studies and 0.842 overall; with the institutional export convention (width 400/level 25 followed by JPEG quality 75), it was 0.929 overall (Table 6). The external pass rate thus depended on how the external images were converted, which is consistent with the gate measuring representation consistency. Among the 288 flagged slices with the submitted conversion, the rules involved were low histogram correlation (183), low entropy (68), histogram distance (53), high extreme-value fraction (47), low Laplacian variance (37) and low edge (1). Histogram-rule flags occurred mainly at the inferior end of the sampled range (thoraco-abdominal level; median normalized position 0.04–0.09 vs. 0.57 for passed slices) with a larger body fraction; low-entropy and extreme-value flags came from 6–7 studies with a smaller body fraction (0.43 vs. 0.56) and a low noise estimate; low-Laplacian flags came from three studies with the smallest pixel spacing (0.56 mm). At the study level, the flag rate was associated with body fraction (Spearman ρ = 0.35) and mean intensity (ρ = 0.35) but not with an image-noise estimate (ρ = −0.03). Flags were concentrated: 47 of 76 studies had at least one flagged slice, five studies had more than half flagged, and five studies contributed 89 of the 288 flags. Manufacturer tags were removed in this public subset, so manufacturer-specific behavior could not be assessed; the pass rate was 0.892 for STANDARD-kernel studies (n = 50) and 0.716 for B-kernel studies (n = 16), which are small and confounded subgroups. Of the 218 image-level PE-positive slices, 204 (93.6%) passed.

Controlled degradations (Table 6) were flagged in 100% of slices for blur, down-sampling and JPEG quality 12, and the flag rate increased monotonically with the severity of intensity remapping (20.3%, 44.5%, 73.9%, 93.4%). Across 1000 patient-block bootstrap replicates of the calibration, blur, down-sampling and heavy compression were flagged in 100% of slices in every replicate and the remapping response was monotone in every replicate, whereas the pass rate on unmodified external images varied widely (2.5–97.5% range of the flag rate 11.5–55.0%), driven mainly by the lower-tail thresholds of Laplacian variance and edge density. Leaving out one calibration patient at a time, the held-out patient’s slice decisions agreed with the full-calibration decisions in 98.4%, 93.4% and 97.2% of slices on average (normal non-contrast, PE contrast, PE non-contrast), and the external pass rate was 0.804–0.851 for 10 of the 11 omissions and 0.698 when one influential patient was omitted. Adding 109 training/validation series to the calibration (41 patients) changed the institutional pass rates little (contrast 0.896 to 0.897; non-contrast 0.892 to 0.919) but changed 8.6–10.7% of individual slice decisions and lowered the external pass rate with the submitted conversion from 0.842 to 0.688 (0.853 with the institutional convention). The absolute pass rate on unmodified images is therefore a calibration- and conversion-dependent quantity, whereas the detection of blur, down-sampling and heavy compression was stable. On the 24 external non-contrast series (Section 3.7), where manufacturer tags were available, the pass rate was 0.802 with the institutional convention (GE 0.920, Siemens 0.660, Toshiba 0.832) and 0.314 with the submitted conversion. Within the Siemens series, the pass rate was 0.900 for the three B30f 1.8 mm series and 0.306 for the three B31f 3.0 mm series (0.657 for two B45f series), and it was 0.832 for the Toshiba FC01 3.0 mm series; flags came mainly from the histogram correlation, histogram distance and Laplacian variance rules. The pass rate therefore varied with the reconstruction more than with the manufacturer, with eight series per manufacturer.

### 3.2. Sealed Test Results

**Figure 5:**
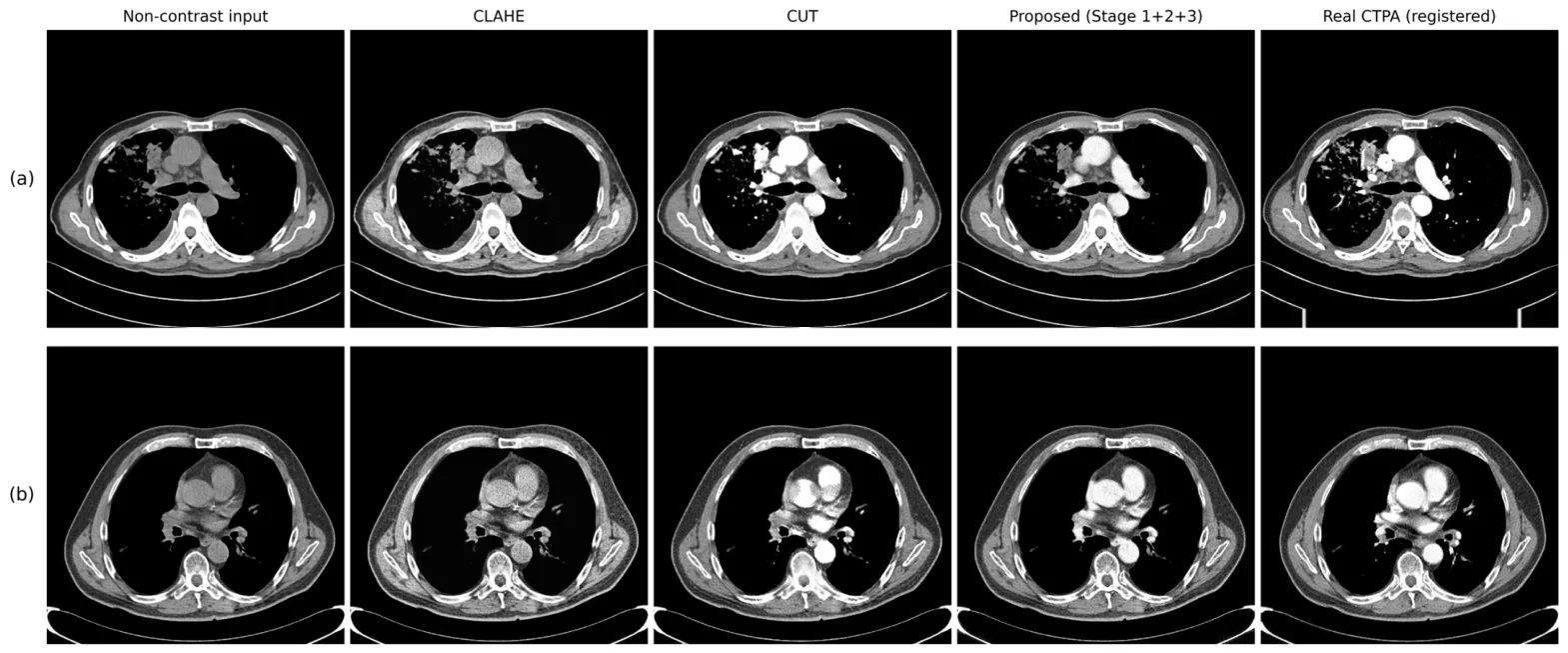
Qualitative comparison with the sealed test cohort. Two representative cases are shown (**a**,**b**). From left to right: acquired non-contrast input, CLAHE, CUT, final Stage 1 + 2 + 3 output, and the corresponding registered real CTPA reference.

The final output (Stage 1 + 2 + 3) and the comparison methods were evaluated on the sealed test cohort of 30 patients, which had not been used for model selection (Table 7, Figure 5). Patient-level paired statistics are presented in Table 8.

Relative to the non-contrast input, the mediastinal intensity distribution distance decreased from 14.92 to 7.19 (median paired difference −8.82, 95% CI −13.32 to −5.09; 26 of 30 patients), the vascular support distribution distance decreased from 42.14 to 15.38 (−21.84, −25.76 to −20.58; 29/30), and the contrast-gap error decreased from 11.00 to 4.25 (−6.50, −9.00 to −4.75; 29/30) (all Holm-adjusted *p* < 0.001). Compared with CLAHE, significant differences were also found in all four distribution and contrast metrics, and all differences were in the direction favoring the proposed method.

In the comparison with CUT, no statistically significant differences were identified in the four distribution and contrast metrics. The vascular support distribution distance was lower for CUT (11.90 vs. 15.38 for the proposed method; paired difference +1.27, 95% CI −0.10 to 5.85; lower for the proposed method in 11 of 30 patients), and the mediastinal distribution distance was lower for the proposed method (7.19 vs. 9.91; −2.40, −4.29 to 0.67; 19 of 30), but neither comparison was significant after Holm correction. These non-significant results were therefore not interpreted as evidence of equivalence between the two methods.

In the input-structure agreement metrics, by contrast, consistent differences from CUT were identified. For the proposed method, EPI was higher (0.779 vs. 0.390 for CUT; +0.383, 95% CI 0.378 to 0.395), the novel-edge rate was lower (0.0111 vs. 0.0201; −0.0089, −0.0137 to −0.0068), and the lost-edge rate was lower (0.0053 vs. 0.0429; −0.0354, −0.0432 to −0.0293). All three metrics were in the direction favoring the proposed method in 30 of 30 patients, with Holm-adjusted *p* < 1 × 10^−4^. Thus, while no statistically detectable difference was identified between CUT and the proposed method in the distribution and contrast metrics, a consistent difference was observed in the extent of structural alteration relative to the input.

Compared with CLAHE, the novel-edge rate was lower for the proposed method in 30 of 30 patients (0.0254 vs. 0.0111, *p* < 1 × 10^−4^), whereas the lost-edge rate was lower for CLAHE in 30 of 30 patients (0.0004 vs. 0.0053, *p* < 1 × 10^−4^). The two edge-based metrics therefore reflect different patterns of structural change, and the novel-edge rate of CLAHE was approximately 2.3 times that of the proposed method. The cohort-level FID was lowest for CUT (CUT 19.4; proposed 22.6); FID is not a patient-level paired metric and was therefore not included in the paired analysis of Table 8. CUT therefore showed lower values in support_emd and FID, and the proposed method showed lower structural alteration; we interpret the comparison as a trade-off between contrast-like distribution and alteration of the input structure rather than as an overall ranking.

The representative cases in Figure 5 complement the quantitative results visually. Compared with the non-contrast input and CLAHE, both CUT and the proposed method showed increased opacification in the vascular and mediastinal regions, and structural change was less pronounced for the proposed method than for CUT.

### 3.3. Stage 3 Component Analysis

The residual blocks of the generator operate at one-quarter of the output spatial resolution and are therefore limited in their ability to retain fine spatial-frequency information directly. In the frequency analysis, the band corresponding to 8 px and finer accounted for only 0.26% of the total power of the residual representation. In contrast, the power ratios of the high-frequency octaves newly introduced after the two decoder up-sampling layers were 0.064 and 0.075. On the basis of these observations, we evaluated the effect of frequency-split composition, which inherits the finest spatial band from the input.

The first component, frequency-split composition (Equation (6)), was compared with and without application (Table 9; the residual refinement was disabled in both conditions). After application, the Nyquist peak changed from 1.019 to 0.937, approaching the input reference value of 0.935, and sharpness changed from 1.227 to 1.017, approaching the reference value of 1.000. The novel-edge rate decreased from 0.0149 to 0.0085 and EPI increased from 0.663 to 0.802. The primary intensity distribution metric, roi_emd, showed no difference (6.95 vs. 6.99, *p* = 0.98).

**Figure 6:**
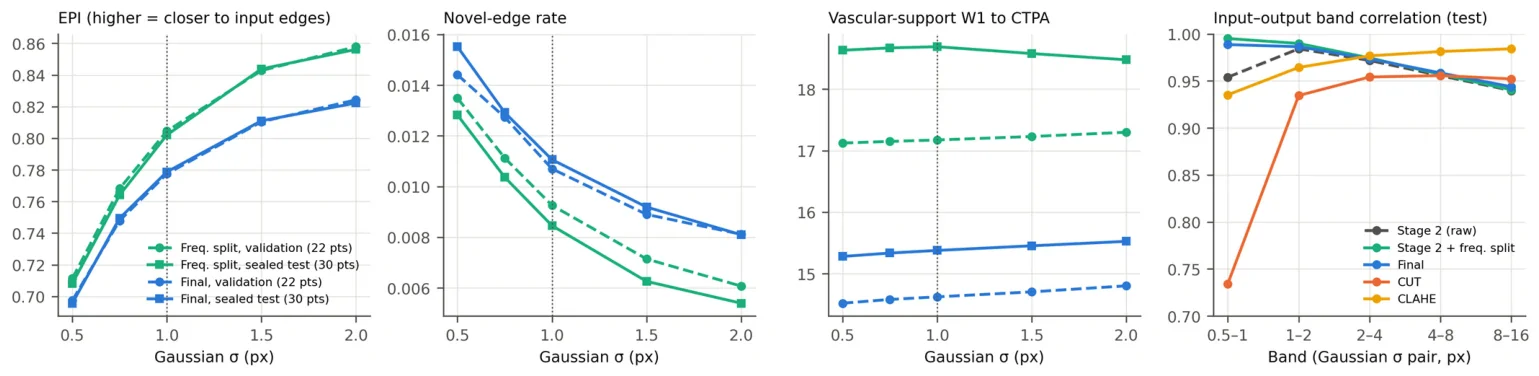
Sensitivity to the Gaussian width σ. (**left**): distribution/contrast and input-structure metrics on the validation and test cohorts; (**right**): input–output correlation of band-pass components for each method. W1, Wasserstein-1 distance, i.e., the earth mover’s distance (EMD); pts, patients.

*Sensitivity to σ:* With the generator and refiner fixed, σ was varied from 0.5 to 2 px on the validation (22 patients, 402 slices) and test cohorts (30 patients, 3428 slices) (Figure 6; Supplementary Table S3). Distribution and contrast metrics changed little across this range (test, final output: roi_emd 7.17–7.26, support_emd 15.28–15.53, gap_error 4.00–4.50), whereas input-structure metrics changed monotonically with σ (EPI 0.696 to 0.822; novel-edge rate 0.0155 to 0.0081; lost-edge rate 0.0058 to 0.0040). The spatial placement of the intensity change was also nearly independent of σ: from σ = 0.5 to 2 px, the mean change in the 2-pixel ring outside the vascular support relative to the change inside it rose from 0.644 to 0.655, and the mean change inside the support fell from 22.65 to 22.39 gray levels (paired differences to σ = 1 in the expected direction in 28–29 of 30 patients for the ring-to-support ratio and 24–28 of 30 for the change inside the support; Supplementary Table S3). σ therefore acts mainly as a structure-alteration control within this range, and larger σ would have lowered structural alteration with little change in the contrast metrics or in where the change is placed; σ = 1 px was retained because it was fixed before the test evaluation and re-selecting it on the test cohort would bias the reported performance.

*Multi-scale agreement:* To examine structure at other spatial scales, we computed the correlation between band-pass (difference-of-Gaussian) components of output and input within the body (Figure 6, right). The composition raised the correlation in the finest band (σ pair 0.5–1 px) from 0.954 (Stage 2) to 0.995; in coarser bands, the output followed the Stage 2 generator (final output 0.986, 0.973, 0.958 and 0.944 for the 1–2, 2–4, 4–8 and 8–16 px bands; Stage 2 alone 0.984, 0.972, 0.955 and 0.940). In the 4–16 px bands, the correlation was similar to CUT (0.952–0.956) and lower than CLAHE (0.981–0.984). Equation (6) therefore protects the finest band; structure at coarser scales is determined by the generator and is not guaranteed by the composition.

Adding the second component, the residual refinement, reduced the vascular support distribution distance from 18.69 to 15.38 relative to the configuration with frequency-split composition alone (29 of 30 patients, Holm-adjusted *p* < 1 × 10^−4^), and the support saturation error from 0.167 to 0.076 (27 of 30 patients, Holm-adjusted *p* < 1 × 10^−4^). The contrast-gap error also decreased from 5.00 to 4.25 (Holm-adjusted *p* < 0.001). No statistically significant difference was identified in the whole-body distribution distance (*p* = 0.13).

*Spatial extent of the refinement:* On the validation cohort, the learned gate averaged 0.090 inside the training support and 0.008 outside its 9 × 9-dilated boundary; the mean absolute correction was 5.00 gray levels inside and 0.32 outside (99th percentile outside, 6.5 gray levels). The correction is thus concentrated on the support but not zero elsewhere, and it contains fine-scale content (high-pass RMS 1.7 gray levels) because the full-resolution gate multiplies the up-sampled residual. The refinement lowered EPI relative to the composition alone (median paired change −0.028, 95% CI −0.033 to −0.020) and the finest-band correlation from 0.995 to 0.989.

### 3.4. Lesion Region Analysis

**Figure 7:**
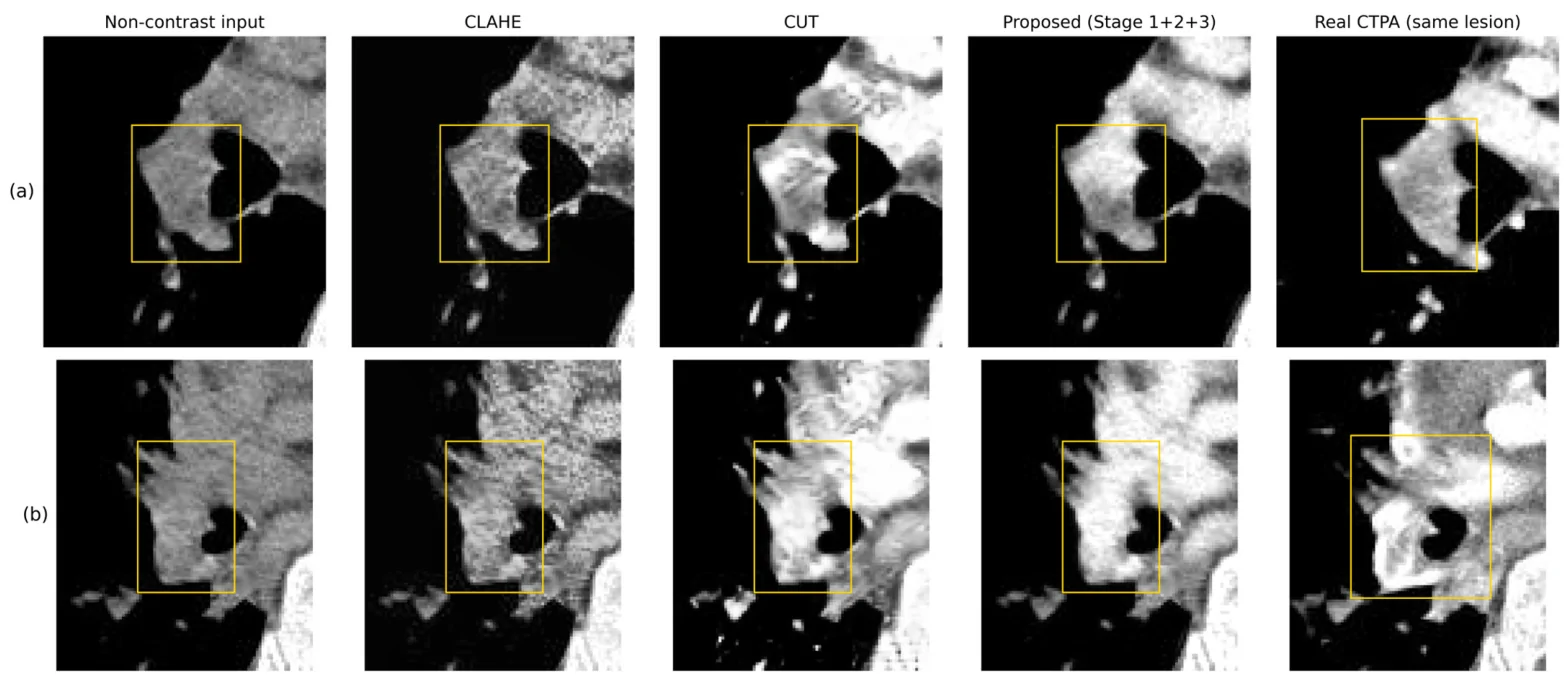
Qualitative comparison of radiologist-annotated lesion regions. Two representative cases are shown (**a**,**b**). From left to right: acquired non-contrast input, CLAHE, CUT, final Stage 1 + 2 + 3 output, and the corresponding real CTPA image showing the same lesion. Yellow boxes indicate the annotated lesion regions used for qualitative comparison. The real CTPA images are provided as visual references and are not used as pixel-wise ground truth for the synthetic outputs.

Local contrast was evaluated within the radiologist lesion boxes: in each non-contrast input, the darkest and brightest thirds of the tissue pixels in a box were fixed as pixel sets, and the difference between the mean intensities of the two sets was computed; the same pixel coordinates were used for every method. The analysis included 248 lesion boxes on 176 annotated non-contrast images (11 patients, 18 unique examinations) and 583 intensity-matched control boxes (Figure 7).

Patient-level lesion-box contrast was 49.2 for the non-contrast input, 80.7 for CLAHE, 82.9 for CUT and 90.6 for the final output; relative to the input, it increased in all 11 patients for the proposed method (median paired change +28.2; Holm-adjusted *p* = 0.005, the smallest attainable with 11 patients). A similar increase occurred in the control boxes (+26.9). The direct test of specificity—the lesion change minus the control change within each patient—was +1.34 gray levels (95% CI −0.62 to 5.92; Wilcoxon *p* = 0.17; positive in 8 of 11 patients), so the increase was not specific to the lesions and is consistent with a general expansion of local intensity differences (Supplementary Table S5, which also reports CLAHE, CUT and Stage 2 and the effect of repeated-study weighting). Between-method differences in lesion-box contrast were not significant. This analysis is not an evaluation of lesion detection; detection is examined in Section 3.5.

The representative cases in Figure 7 show the same quantitative tendency visually. In the corresponding lesion region of the real CTPA, a relatively hypoattenuating area was observed within an opacified vessel; in the proposed output, the intensity of the surrounding vascular region increased at the same location; a relatively dark area was visible in this example, but the quantitative analysis above did not show a lesion-specific effect. In the non-contrast input, the local intensity difference at that location was less pronounced. This qualitative comparison is intended to complement the local contrast analysis visually and does not indicate that the embolus itself was reproduced in the synthesized image.

### 3.5. Effect on an Independent CTPA-Trained PE Model

*Validity of the test and of the format change:* On real CTPA, the model separated PE from normal examinations on the original DICOM (AUROC 0.887, 95% CI 0.732–1.000) and on display-rendered CTPA passed through the same display-to-HU conversion as every enhanced input (0.854, 0.693–1.000); it predicted no PE volume in the median normal examination and intersected 80–83% of the radiologist boxes (patient median) (Table 10). The conversion from 8-bit display images therefore did not by itself prevent detection.

*Non-contrast input:* On non-contrast CT, the model labeled large parts of the non-opacified pulmonary arteries as PE in both groups (median 9.6 mL in PE and 12.0 mL in normal examinations on display images), and discrimination did not differ from chance on display images or on the original DICOM (AUROC 0.392, 95% CI 0.197–0.639, and 0.418, 0.220–0.656).

*What the enhancement changed:* After enhancement, the PE volume predicted in normal patients decreased from a median of 12.0 to 1.7 mL (decrease in 23 of 25 patients; Holm-adjusted *p* < 0.001). It decreased to 4.8 mL with CUT (19 of 25; *p* = 0.003) and did not change with CLAHE (12.3 mL; *p* = 0.85), and in an exploratory comparison the final output was lower than CUT (median paired difference −2.79 mL, lower in 19 of 25 patients; Wilcoxon *p* = 0.002). Across all thresholds, only the two outputs of the proposed pipeline reached operating points below 2.9 mL per normal examination (0.75 mL for the final output and 0.63 mL for Stage 2 with composition), whereas the input, CLAHE and the original non-contrast DICOM did not fall below 6.55 mL and CUT not below 2.94 mL (Supplementary Table S9). The reduction concerned the extent of false-positive labeling rather than the number of candidate regions (median 3.0 to 2.0 components of at least 100 mm^3^ per normal examination; *p* = 0.13).

*What it did not change:* Detection did not improve. Study-level AUROC was 0.470 (0.279–0.699) for the final output, 0.521 for CUT and 0.434 for CLAHE (final − input +0.078, −0.119 to 0.276), and it remained between 0.312 and 0.530 for every representation at every threshold. Lesion-box sensitivity decreased from 0.58 to 0.15 (decrease in 8 of 11 patients; Holm-adjusted *p* = 0.043); at a matched false-positive burden of 10 mL per normal examination, it was 0.581 (0.405–0.723) for the input and 0.125 (0.052–0.255) for the final output. The result did not depend on the study score or on the export window of the PE examinations (Supplementary Table S9), and it was replicated in 28 further PE examinations of 16 patients not used to train Stages 2–3, including the sealed test patients (final output 0.474, 0.304–0.680; input 0.533; real CTPA 0.785; Supplementary Table S9). The reasons are discussed in Section 4.4.

### 3.6. Robustness Analyses

*Repeated training:* Four additional runs (seeds 101, 202, 303, 404) retrained both learned stages with the submitted configuration (CycleGAN 13 epochs, refiner 1 epoch; the original epoch search was not repeated). Test cohort medians across the five runs were roi_emd 7.19–7.61, support_emd 13.52–19.78, gap_error 2.75–6.25, EPI 0.779–0.819 and lost-edge rate 0.0049–0.0200 (Supplementary Table S6). In every run, all four distribution and contrast metrics improved relative to the non-contrast input, and EPI, novel- and lost-edge rates were more favorable than for CUT (all *p* < 0.05); CUT had a lower support_emd in every run (significant in 2 of 4). Two runs (seeds 202 and 303) did not satisfy the refiner’s validation EPI criterion (decrease of 0.044 and 0.036 against an allowed 0.03); these were the two runs with higher lost-edge rates on the test cohort (0.018 and 0.020, vs. 0.0049–0.0053 for the other runs; 0.0067 before refinement for seed 303), so the validation criterion identified them before testing, and under the selection protocol they would not have been deployed. The refiner is therefore the component most sensitive to initialization.

*Adjacent-slice consistency:* On 3375 pairs of consecutive display images (44 test examinations), the patient-median absolute change in the correction field (output minus input) between adjacent slices was 5.01 gray levels for the final output, 4.55 for the composition alone, 10.74 for CUT and 8.28 for CLAHE (final − CUT −5.36, 95% CI −5.70 to −5.04). Because consecutive display images carry neither the slice order nor the spacing, the measurement was repeated on the 74 non-contrast volumes built for the downstream analyses, ordered by DICOM image position (49 patients; slice spacing 3 mm in 71, 5 mm in 2 and 2 mm in 1 examination): the change was 5.43 gray levels for the final output, 4.66 for the composition alone, 11.44 for CUT and 8.96 for CLAHE (final − CUT −5.74, 95% CI −6.18 to −5.25, lower in 49 of 49 patients; final − CLAHE −3.24, −3.89 to −2.73; Wilcoxon *p* < 10^−13^ for both; Supplementary Table S7). Within the mediastinal ROI, the corresponding values were 9.18 and 15.69 gray levels for the final output and CUT. Supplementary Figure S2 shows a coronal reformat of the examination with the median value; no 3D reference with paired CTPA was available.

*Failure cases:* Objectively selected cases are shown in Supplementary Figure S1 (lowest EPI 0.675, highest novel-edge rate 0.073, highest lost-edge rate 0.067 among 3428 test slices).

### 3.7. External Feasibility of the Complete Pipeline

For the 24 external non-contrast series from three manufacturers, the gate passed 80.2% of slices, and 2004 gate-passed central slices were enhanced. After Stage 2–3, the mediastinal distribution distance to the external CTPA reference decreased from 15.52 to 8.66 (median paired change −7.76, 95% CI −9.57 to −3.83; lower in 22 of 24 patients; Wilcoxon *p* = 0.0002), the whole-body distance decreased from 19.09 to 13.50 (−4.30, −6.01 to −3.18; *p* = 0.0001), and the contrast-gap error decreased from 13.5 to 6.0 (−7.75, −9.50 to −6.00; *p* = 0.015) (Table 11). The mediastinal distance decreased for each manufacturer (GE 16.50 to 9.82, Siemens 12.38 to 9.58, Toshiba 17.18 to 5.98). Input-structure metrics were close to the internal values (EPI 0.777 vs. 0.779 internal; novel-edge 0.0134 vs. 0.0111; lost-edge 0.0036 vs. 0.0053). Compared with CUT, EPI was higher (+0.406, 0.371 to 0.439; 24 of 24 patients), the novel- and lost-edge rates were lower (−0.0087, −0.0198 to −0.0051, 21 of 24; −0.0285, −0.0410 to −0.0213, 24 of 24), and the mediastinal distribution distance was lower (−3.11, −7.11 to −1.73; *p* = 0.001), whereas the whole-body distance was lower for CUT (+1.59, 1.23 to 2.62; *p* = 0.10) and the gap error did not differ (*p* = 0.44). The 16 series analyzed before the eight Toshiba series were added gave the same directions (Supplementary Table S8). No paired external CTPA was available, so this analysis shows the direction of the effects on unseen scanners, not their accuracy. The distribution shift was smaller than internally (roi_emd to the same reference: internal 19.80 to 6.44). For these external volumes, the independent PE model predicted a median PE volume of 11.9 mL on the raw input and 4.4 mL after enhancement (decrease in 19 of 24 patients, including 8 of 8 Toshiba series; Wilcoxon *p* < 0.001; PE status not annotated), a smaller reduction than in the internal normal patients (12.0 to 1.7 mL), consistent with the smaller external distribution shift.

## 4. Discussion

### 4.1. Principal Findings

In this study, we evaluated a three-stage contrast-like enhancement framework for display-domain non-contrast chest CT that combines an input representation-consistency gate, unpaired global translation, and structure-constrained local refinement. On the sealed test cohort, the proposed method improved the intensity distribution and contrast metrics relative to the non-contrast input and to CLAHE. Compared with CUT, no statistically significant differences were detected in the distribution and contrast metrics; EPI, the novel-edge rate, and the lost-edge rate, however, were in the direction favoring the proposed method in all 30 patients (all *p* < 1 × 10^−4^). The same pattern was observed in the external feasibility analysis of the complete pipeline, which had no paired CTPA (Section 3.7). The principal result is therefore a trade-off: a contrast-like intensity distribution comparable to CUT with lower alteration of the input edges. An independent 3D PE model trained on CTPA showed both what this representation provides and where it stops: normal arteries were largely no longer labeled as embolus, whereas embolus detection, which depends on contrast that non-contrast acquisitions do not contain, did not change (Section 4.4).

### 4.2. Relation to Previous Work

Previous studies have reported that GANs can generate synthetic contrast enhancement from non-contrast chest CT [19], and CycleGAN enables unpaired domain translation in settings where registered paired images are scarce [21]. Producing an output close to the target image distribution and retaining the anatomical structure of the input patient, however, do not necessarily coincide. In the present study as well, CUT had the lowest cohort-level FID but showed larger changes than the proposed method in the input-structure agreement metrics. FID is a set-level metric for which patient-level paired comparison does not hold, and it is therefore difficult to use as a basis for judging structural alteration in an individual study.

Because display-domain images are provided with limited information on HU, window/level, and bit depth [14,15,16], it is difficult to set the reproduction of a real CTPA as the goal of the translation. In this study, the objective was to generate a contrast-like representation while retaining the structure of the acquired image, and the generation quality of display-domain medical images needs to be assessed by considering patient-level input-structure agreement metrics together with, rather than solely on the basis of, distribution-based metrics.

### 4.3. Stage 3 Components

The Stage 3 ablation showed the roles of the two components concretely. After frequency-split composition was applied, the Nyquist peak and sharpness approached the input reference, EPI increased, and the novel-edge rate decreased, whereas no significant change was observed in roi_emd. Subsequently adding the residual refinement reduced the distribution and contrast-related error within the vascular support, while no significant change appeared in the whole-body distribution. The two components may thus be interpreted as playing complementary roles: inheritance of the finest spatial band and refinement of the residual signal around the vascular support.

The property that the finest band of the output is inherited from the acquired image follows from Equation (7) rather than from an experiment: the acquired image is modified only by a Gaussian-smoothed difference. This construction does not by itself preserve structure at coarser scales, and the multi-scale analysis showed that agreement with the input in bands above ~2 px is set by the generator. The sensitivity analysis showed that σ controls the balance between structural alteration and generator content with little effect on the contrast metrics in the range 0.5–2 px. The refinement adds a correction concentrated on, but not confined to, the vascular support.

### 4.4. Scope of Contrast-Like Harmonization for PE AI

The downstream analysis tested one specific use: whether enhanced 2D display images can take the place of CTPA as the input of an existing 3D PE detector trained on CTPA volumes. Two format differences are part of this setting—slice-wise 2D processing and the conversion from 8-bit display values to Hounsfield units—and neither was what limited the result: the detector worked on display-rendered CTPA passed through the same conversion (AUROC 0.854), the enhancement varied less between adjacent slices than CUT or CLAHE (Section 3.6), and the detector could not separate PE from normal examinations on the original 3D non-contrast DICOM either (AUROC 0.418). The limit therefore lies in the non-contrast acquisition rather than in the processing. On non-contrast CT an embolus and a normal unopacified artery present the same feature, and a translation learned without lesion information cannot place embolus–blood contrast that its input does not contain. Consistent with this, local contrast increased similarly at lesion and control positions (Section 3.4), and in an exploratory analysis the lowest reachable false-positive volume and the highest reachable sensitivity gave the same ordering across all six representations (Spearman ρ = 1.000, n = 6).

Within this scope, the harmonization changed how the detector read non-contrast CT. On raw non-contrast images, it labeled much of the unopacified pulmonary arteries as embolus; after enhancement, the volume labeled in normal patients fell by 85%, and only the outputs of the proposed pipeline reached operating points below 2.9 mL per normal examination. This matters when non-contrast studies enter data processed by CTPA-trained models. The enhanced images should not, however, replace CTPA as the input of CTPA-trained detectors, and whether a detector trained on harmonized non-contrast CT would benefit could not be tested because lesion-level annotations on non-contrast CT were available for only 11 patients. The contribution of the pipeline to PE AI therefore lies in data preparation: harmonizing the representation of display-domain non-contrast CT with limited structural alteration.

### 4.5. Representation-Consistency Gate and External Evaluation

Before translation, the Stage 1 representation-consistency gate uses a DICOM-verified reference to screen display-domain inputs whose representation departs from the calibrated display convention. A decrease in performance has been reported when a model trained on public data was applied to images from another institution [11], so that a procedure for handling the heterogeneity of input conditions before translation has practical value.

The gate detected blur, down-sampling and heavy compression in every external image, and this held across calibration resamples and after calibration expansion. Its pass rate on unmodified images, in contrast, depended on the display conversion and on the calibration sample (external pass rate 0.842 with the submitted conversion, 0.929 with the institutional convention; 0.688–0.853 with the expanded calibration). The flags on unmodified external images were associated with slice position, field of view and reconstruction smoothness rather than with noise. For deployment, the thresholds should therefore be interpreted as a local representation convention: they identify inputs that differ from the calibrated display convention or carry common degradations, and they would need recalibration on a representative sample when the export convention of a new site differs.

### 4.6. Limitations and Future Work

The scope of this study is the conversion of the representation of display-domain images into a contrast-like representation. In 8-bit display-domain images, the true HU values and the original window/level settings cannot be recovered, and a weakly registered CTPA cannot be regarded as pixel-wise ground truth either. The proposed output should therefore not be interpreted as a quantitative CT image with recovered HU values or as a diagnostically equivalent CTPA.

Stage 2 and 3 were developed on single-center retrospective data; external testing covered the complete pipeline only as a feasibility analysis on 24 non-contrast scans from three manufacturers without paired CTPA, and Stage 1 on 76 external CTPA studies. The gate calibration relied on 36 patients, and its absolute pass rate was calibration- and conversion-dependent. The samples were 30 patients for the test, 11 patients for the lesion analysis and 11 plus 16 PE patients for the downstream analyses, so the non-significant comparisons with CUT cannot be read as equivalence. The downstream analysis used one independent model and small PE cohorts, only one of which had lesion annotations; it showed no improvement in detection and does not address detectors trained on non-contrast CT. Stages 2 and 3 process each slice independently; inter-slice consistency was measured on DICOM-ordered volumes, but without a paired 3D CTPA reference. Future work includes multicenter non-contrast data with paired CTPA, detectors trained on PE-labeled non-contrast data, and a blinded reader study. Reporting followed [17,18].

## 5. Conclusions

This study proposed a quality-gated contrast-like enhancement pipeline for display-domain non-contrast chest CT in settings where original DICOM files and registered NCT–CTPA pairs are limited. The final output improved the distribution and contrast metrics relative to the non-contrast input and to CLAHE; compared with CUT, it did not differ significantly in distribution matching on the internal test cohort and altered the input edges less, a pattern observed in all five training runs and in a feasibility analysis of the complete pipeline on external non-contrast CT from three manufacturers without paired CTPA, with lower variation in the correction between adjacent slices than CUT. Tested on external CTPA, the input gate responded consistently to blur, down-sampling and heavy compression, while its pass rate on unmodified images depended on the display conversion. Local contrast increased at annotated lesions without lesion specificity, and with an independent 3D PE model trained on CTPA the enhanced images reduced the labeling of normal arteries as embolus without improving detection, which requires embolus–blood contrast that non-contrast acquisitions do not contain. The pipeline is therefore suited to harmonizing the representation of display-domain non-contrast CT with limited structural alteration, and its role in PE AI lies in data preparation rather than in replacing CTPA for detection.

## Figures and Tables

**Table 1:** Patient-level data split of the hospital display-domain cohort.

Split	Patient	Study	Non-Contrast	Contrast	Role
Train	145	243	27,815	34,508	Stage 2 and 3 Training
Validation	22	35	3963	4857	Model and Epoch Selection
Test (sealed)	30	44	4835	6403	Final Evaluation
Lesion	11	43	5016	5736	For Lesion Evaluation Only
Total	208	365	41,629	51,504	
DICOM-verified calibration (normal)	25 †	32	2560 ‡	–	Stage 1 reference; downstream PE-negative group
DICOM-verified calibration (PE)	11 §	18	1584 ‡	1584 ‡	Stage 1 reference
Calibration expansion (sensitivity only)	30 ¶	55/54 series	4318 ‡	4397 ‡	Stage 1 sensitivity analysis
External RSNA-STR (CTPA)	76 studies	76	–	1824	Stage 1 external test; external CTPA reference
External LIDC-IDRI (NCT)	24	24	3095	–	Complete-pipeline external feasibility

† not part of the 208-patient cohort. ‡ sampled images (≤80 per series). § same patients as the lesion cohort. ¶ training/validation patients only. Merged with the original calibration, the expanded profiles contained 5902 non-contrast and 5981 contrast images.

**Table 2:** Preprocessing parameters.

Parameter	Value
HU conversion	RescaleSlope/RescaleIntercept (DICOM only)
Lung window (center/width)	−600/1500
Mediastinal window (center/width)	40/400
Vascular (PE) window (center/width)	100/700
Resize	512 × 512
Bit-depth	8-bit
Intensity normalization	[−1, 1] (Stage 2 CycleGAN input); [0, 1] (Stage 3 refiner input); [0, 255] (display and metrics)
Channels	Single (grayscale)

**Table 3:** Agreement between display images and the same DICOM instance, series means (identified window: series-specific width/level from grid search).

Group	*n* (Series)	Window	SSIM ↑	PSNR (dB) ↑	Histogram Distance ↓	Entropy Diff ↓	Best Conventional Window (Series)
Normal, non-contrast	32	Lung/Mediastinal/Vascular	0.189/0.980/0.982	7.8/30.0/31.0	95.07/4.20/3.55	2.320/0.041/0.082	Vascular 28, Mediastinal 4
		Identified	0.998	55.4	0.19	0.004	
PE, contrast	18	Lung/Mediastinal/Vascular	0.177/0.913/0.878	8.1/29.1/22.0	89.86/4.87/10.38	1.873/0.343/0.400	Mediastinal 16, Vascular 2
		Identified	0.928	33.9	1.61	0.374	
PE, non-contrast	18	Lung/Mediastinal/Vascular	0.184/0.933/0.908	7.8/30.6/24.9	95.30/3.92/7.39	1.881/0.307/0.450	Mediastinal 15, Vascular 3
		Identified	0.946	36.6	0.91	0.338	

↑ and ↓ indicate that higher and lower values, respectively, correspond to closer agreement with the DICOM rendering.

**Table 4:** QC-pass rate across the hospital display-domain cohort, by split.

Split	Slice	Non-Contrast Pass Rate	Contrast Pass Rate
Train	62,323	0.877	0.885
Validation	8820	0.953	0.943
Test (sealed)	11,238	0.900	0.891
Lesion	10,752	0.920	0.923
Total	93,133	0.892	0.896

**Table 5:** QC-pass versus QC-flag slices: agreement with the same DICOM instance in the identified window; patient medians of per-patient means, paired Wilcoxon, Holm over 18 tests.

Group (Paired Patients)	Label	SSIM ↑	PSNR ↑	MAE ↓	Histogram Distance ↓	Extreme-Value Fraction Diff ↓	Entropy Diff ↓
Normal, non-contrast (19)	QC-pass	0.9997	56.0	0.18	0.18	0.0001	0.002
	QC-flag	0.9997	55.0	0.21	0.21	0.0001	0.002
	Holm *p*	1.00	1.00	1.00	1.00	1.00	0.87
PE, contrast (8)	QC-pass	0.971	35.6	2.08	0.33	0.040	0.331
	QC-flag	0.970	32.5	2.03	0.35	0.034	0.287
	Holm *p*	1.00	1.00	1.00	1.00	1.00	1.00
PE, non-contrast (9)	QC-pass	0.971	36.9	1.91	0.29	0.038	0.313
	QC-flag	0.971	39.2	1.53	0.27	0.028	0.258
	Holm *p*	1.00	1.00	1.00	1.00	0.14	0.33

↑ and ↓ indicate that higher and lower values, respectively, correspond to closer agreement with the DICOM rendering.

**Table 6:** Gate response on the external cohort (RSNA-STR, 1824 slices/76 studies).

Condition	Injection	Flag Rate, Submitted Calibration	2.5–97.5% over 1000 Calibration Resamples	Flag Rate, Expanded Calibration (41 Patients)	Main Rules
Unmodified conversion	W700/L100	15.8%	11.5–55.0%	31.3%	Low histogram correlation, low entropy
Unmodified conversion	W400/L25 + JPEG q75	7.1%	–	14.7%	Histogram distance
Blur	Gaussian radius 2.0 px	100.0%	100.0–100.0%	100.0%	Low Laplacian variance, low edge
Down-sampling	512→128→512, bilinear	100.0%	100.0–100.0%	100.0%	Low Laplacian variance, low edge
Compression	JPEG quality 12	100.0%	100.0–100.0%	100.0%	Low histogram correlation
Remapping, width 195	[30, 225]→[0, 255]	20.3%	13.8–40.0%	23.1%	Low entropy, high extreme-value fraction
Remapping, width 165	[45, 210]→[0, 255]	44.5%	37.3–64.9%	24.0%	Low entropy, high extreme-value fraction
Remapping, width 135	[60, 195]→[0, 255]	73.9%	68.5–86.9%	54.2%	Low entropy, high extreme-value fraction
Remapping, width 100	[80, 180]→[0, 255]	93.4%	91.6–98.4%	85.2%	Low entropy, high extreme-value fraction

**Table 7:** Quantitative comparison on the sealed test cohort (n = 30 patients).

Method	Roi_emd ↓	Body _emd ↓	Support _emd ↓	Gap _error ↓	EPI ↑	Sharpness	Novel Edge ↓	Lost Edge ↓	FID ↓
Non-contrast (input)	14.92	12.04	42.14	11.00	1.000	1.000	0	0	24.3
CLAHE	13.61	10.51	23.72	17.25	0.778	1.762	0.0254	0.0004	36.4
CUT	9.91	6.11	11.90	7.25	0.390	1.174	0.0201	0.0429	19.4
Proposed (stage 1 + 2 + 3)	7.19	4.84	15.38	4.25	0.779	1.079	0.0111	0.0053	22.6

↑ and ↓ indicate that higher and lower values, respectively, are favorable.

**Table 8:** Paired statistics of the final output (Stage 1 + 2 + 3) on the sealed test cohort (n = 30, paired Wilcoxon, Holm-adjusted). Second line of each cell: 95% patient-bootstrap confidence interval of the median paired difference (20,000 resamples); for the cells marked ^2^, the interval of the value itself.

Metric	vs. Non-Contrast	vs. CLAHE	vs. CUT
roi_emd ↓	−8.82 (26/30, *p* = 2 × 10^−4^) [−13.32, −5.09]	−5.75 (27/30, *p* < 1 × 10^−4^) [−6.59, −3.81]	−2.40 (19/30, ns) [−4.29, 0.67]
body_emd ↓	−5.98 (28/30, *p* < 1 × 10^−4^) [−8.68, −4.66]	−4.60 (26/30, *p* < 1 × 10^−4^) [−6.43, −2.91]	−0.26 (17/30, ns) [−1.37, 0.34]
support_emd ↓	−21.84 (29/30, *p* < 1 × 10^−4^) [−25.76, −20.58]	−6.95 (29/30, *p* < 1 × 10^−4^) [−9.57, −5.41]	+1.27 (11/30, ns) [−0.10, 5.85]
gap_error ↓	−6.50 (29/30, *p* < 1 × 10^−4^) [−9.00, −4.75]	−12.00 (28/30, *p* < 1 × 10^−4^) [−15.50, −9.50]	−1.00 (18/30, ns) [−3.25, 0.25]
EPI ↑	−0.221 (*p* < 1 × 10^−4^) ^1^ [−0.233, −0.209]	+0.008 (21/30, ns) [0.001, 0.016]	+0.383 (30/30, *p* < 1 × 10^−4^) [0.378, 0.395]
sharpness → 1	+0.079 (*p* < 1 × 10^−4^) ^1^ [0.075, 0.086]	−0.681 (30/30, *p* < 1 × 10^−4^) [−0.821, −0.556]	−0.090 (20/30, ns) [−0.160, −0.0005]
novel edge ↓	0.0111 ^2^ [0.0086, 0.0161]	−0.0139 (30/30, *p* < 1 × 10^−4^) [−0.0209, −0.0097]	−0.0089 (30/30, *p* < 1 × 10^−4^) [−0.0137, −0.0068]
lost edge ↓	0.0053 ^2^ [0.0037, 0.0076]	+0.0049 (0/30, *p* < 1 × 10^−4^) [0.0032, 0.0071]	−0.0354 (30/30, *p* < 1 × 10^−4^) [−0.0432, −0.0293]

^1^ The non-contrast input is the self-reference point for EPI and sharpness (1.000 for each); these two cells therefore indicate the extent of structural alteration relative to the input rather than the magnitude of an improvement. ^2^ The non-contrast input is 0 by definition for novel edge and lost edge; these two cells are therefore absolute values for the proposed method rather than differences. ↑ and ↓ indicate that higher and lower values, respectively, are favorable; → 1 indicates that values closer to 1 (no change in sharpness relative to the input) are favorable.

**Table 9:** Ablation of the frequency-split component of Stage 3 on the sealed test cohort. The residual refinement component is disabled in both arms.

Method	Roi_emd	Nyquist Peak	Novel Edge	EPI	Sharpness
Stage 2 output (no synthesis)	6.95	1.019	0.0149	0.663	1.227
Frequency-split synthesis	6.99	0.937	0.0085	0.802	1.017
Non-contrast (reference)		0.935	0	1.000	1.000

**Table 10:** Independent 3D PE model trained on CTPA, applied to 18 PE and 32 normal examinations.

Input	AUROC (95% CI)	Predicted PE Volume, Normal (Median mL)	Normal Exams with ≥1 mL	Lesion-Box Sensitivity (Patient Median)
Non-contrast, true HU	0.418 (0.220–0.656)	10.8	100%	0.54
Non-contrast, display	0.392 (0.197–0.639)	12.0	100%	0.58
CLAHE	0.434 (0.226–0.687)	12.3	100%	0.62
CUT	0.521 (0.326–0.733)	4.8	88%	0.66
Stage 2 + frequency split	0.491 (0.309–0.724)	1.7	66%	0.15
Final (Stage 1 + 2 + 3)	0.470 (0.279–0.699)	1.7	66%	0.15
Real CTPA, display	0.854 (0.693–1.000)	0.0	9%	0.83
Real CTPA, true HU	0.887 (0.732–1.000)	0.0	9%	0.80

**Table 11:** External non-contrast CT (LIDC-IDRI, 24 patients: 8 GE, 8 Siemens, 8 Toshiba; reference: RSNA-STR CTPA, identical conversion). Patient medians.

Method	Roi_emd ↓	Body_emd ↓	Gap_error ↓	EPI ↑	Sharpness	Novel Edge ↓	Lost Edge ↓
Non-contrast input	15.52	19.09	13.5	1.000	1.000	0	0
CLAHE	12.02	12.31	13.5	0.754	1.946	0.0237	0.0003
CUT	12.40	12.19	6.3	0.369	1.214	0.0231	0.0344
Stage 2 + frequency split	8.08	14.43	9.0	0.814	1.016	0.0094	0.0036
Final (Stage 1 + 2 + 3)	8.66	13.50	6.0	0.777	1.109	0.0134	0.0036

↑ and ↓ indicate that higher and lower values, respectively, are favorable.

## Data Availability

The datasets used and analyzed in this study are not publicly available because they are not public-use data and were provided under permission for research purposes only. Institutional policies, patient privacy protections, and the conditions of institutional review board approval restrict public disclosure and external sharing. Code for the revision analyses and summary tables are available from the corresponding author; the public datasets (RSNA-STR PE CT, LIDC-IDRI) and the open-weight PE segmentation model are available from their original sources.

## Supplementary Materials

The following supporting information can be downloaded at: https://www.mdpi.com/article/10.3390/bioengineering13101145/s1, Table S1: Stability of the Stage 1 gate calibration; Table S2: Characteristics of the external images flagged by the gate; Table S3: Sensitivity to the Gaussian width σ; Table S4: Patient-level paired effects on the test cohort; Table S5: Lesion versus intensity-matched control boxes; Table S6: Repeated training runs; Table S7: Change in the correction between adjacent slices on DICOM-ordered volumes; Table S8: External non-contrast CT, complete pipeline; Table S9: Independent 3D PE model trained on CTPA; Figure S1: Objectively selected adverse slices; Figure S2: Coronal reformat of the examination with the median inter-slice change.

## Abbreviations

The following abbreviations are used in this manuscript:

CTPA	Computed tomography pulmonary angiography
NCT	Non-contrast computed tomography
PE	Pulmonary embolism
HU	Hounsfield unit
DICOM	Digital Imaging and Communications in Medicine
QC	Quality control
ROI	Region of interest
EMD	Earth mover’s distance
EPI	Edge preservation index
FID	Fréchet inception distance
SSIM	Structural similarity index
PSNR	Peak signal-to-noise ratio
MAE	Mean absolute error
CLAHE	Contrast-limited adaptive histogram equalization
AUROC	Area under the receiver operating characteristic curve
CUT	Contrastive unpaired translation
LIDC-IDRI	Lung Image Database Consortium and Image Database Resource Initiative
RSNA-STR	Radiological Society of North America–Society of Thoracic Radiology (pulmonary embolism CT dataset)
