# Supplementary material for: Quality-Gated CycleGAN with Frequency-Split Composition for Structure-Constrained Contrast-Like Enhancement of Non-DICOM Chest CT: External Feasibility and Implications for Pulmonary Embolism AI
Source: Bioengineering (Basel). 2026 Sep 30;13(10):1145. doi: 10.3390/bioengineering13101145 (PMC13701485; doi:10.3390/bioengineering13101145)
Supplement: Supplementary file 1 [file bioengineering-13-01145-s001.zip › bioengineering-4566718-supplementary.pdf]

Supplementary Material

Quality-Gated CycleGAN with Frequency-Split Composition for Structure-Constrained Contrast-Like Enhancement of Non-DICOM Chest CT (bioengineering-4566718, revised)

Supplementary Methods

Pre-specification. The downstream analysis with the independent PE model, the external feasibility analysis and the additional calibration, σ and seed analyses were specified in written protocols before their outcomes were computed. For the downstream analysis with the independent PE model (Section 2.8.1), a sanity run on two true-HU examinations showed that, on non-contrast CT, the model labels large parts of the non-opacified pulmonary arteries as PE and therefore intersects most lesion boxes; the primary end point was therefore moved from lesion-box sensitivity to study-level AUROC before any enhanced, CLAHE or CUT input was processed. The re-analysis over the full threshold range, the spatial placement of the change as a function of σ, the replication cohort and the inter-slice analysis on DICOM-ordered volumes were each registered before computation. Two comparisons were computed after the corresponding results had been seen and are labelled exploratory: the false-positive volume of the final output against CUT, and the rank correlation across representations between the lowest reachable false-positive volume and the highest reachable sensitivity.

External selection rule. LIDC-IDRI series were accepted as non-contrast when the 90th percentile of mediastinal HU on mid-thoracic slices was below 100 HU and no contrast agent was recorded, up to eight per manufacturer, in patient-ID order. After screening and before any processing, a contrast-agent tag that explicitly recorded no IV contrast ("W/O IV CONTRAST") was counted as no agent recorded; this added eight Toshiba series and changed no other decision. The results for the 16 series analysed before this are given in Table S8c.

Statistics. Unless stated otherwise, values are patient medians; paired differences are medians of within-patient differences with 95% percentile bootstrap intervals over patients (20,000 resamples) and Wilcoxon signed-rank p values.

Table S1. Stability of the Stage 1 gate calibration (Reviewer 1, Comment 1)

(a) Designed flag rate. Each of the six one-sided rules excludes 2.5% of the reference images, so the gate flags part of its own reference set by construction.

Reference profile	Series (patients)	Reference slices	In-sample pass rate
Normal non-contrast	32 (25)	2,560	0.892
Pe contrast	18 (11)	1,584	0.912
Pe non-contrast	18 (11)	1,584	0.914

(b) Unmodified external images across 1,000 patient-block calibration resamples (template and thresholds re-estimated each time; RSNA-STR, 1,824 slices, submitted conversion). The rows show the fraction of slices flagged by each rule; the variation of the total comes mainly from the Laplacian-variance and edge rules. The degradation rows of Table 6 give the same resamples for blur, down-sampling, compression and remapping.

Rule	2.5th percentile	Median	97.5th percentile
All rules (total flag rate)	0.115	0.202	0.550
Low entropy	0.031	0.039	0.105
High extreme-value fraction	0.014	0.026	0.073
Low edge density	0.000	0.001	0.243
Low Laplacian variance	0.017	0.020	0.364
Histogram distance	0.003	0.031	0.123
Low histogram correlation	0.040	0.106	0.191

(c) Leave-one-patient-out re-estimation (one of the 11 calibration patients omitted at a time; decisions for the held-out patient compared with the full calibration).

Reference profile	Pass rate, full calibration	Pass rate, held-out patient	Decision agreement
Normal non-contrast	0.876	0.860	0.984
Pe contrast	0.927	0.860	0.934
Pe non-contrast	0.907	0.880	0.972

Reference profile	Pass rate, full calibration	Pass rate, held-out patient	Decision agreement

External pass rate (submitted conversion) with one calibration patient omitted: 0.804–0.851 for 10 of the 11 omissions and 0.698 for one influential patient.

(d) Calibration expanded from 11 to 41 patients (109 further DICOM-verified series from training and validation patients; test and lesion patients excluded).

Phase	Series (patients)	Institutional pass rate	Slice decisions unchanged	External pass rate, submitted conversion
Contrast	18 (11) → 73 (41)	0.896 → 0.897	0.893	0.842 → 0.688
Non-contrast	18 (11) → 72 (41)	0.892 → 0.919	0.914	–

(e) External pass rate by display conversion (the same 1,824 RSNA-STR DICOM slices rendered in three ways).

Calibration	Conversion	All slices	PE studies	PE-negative studies
Submitted (11 patients)	Width 400 / level 25	0.822	0.840	0.804
Submitted (11 patients)	Width 400 / level 25 + JPEG q75 (institutional)	0.929	0.933	0.925
Submitted (11 patients)	Width 700 / level 100 (submitted)	0.842	0.884	0.798
Expanded (41 patients)	Width 400 / level 25	0.741	0.756	0.725
Expanded (41 patients)	Width 400 / level 25 + JPEG q75 (institutional)	0.853	0.859	0.847

Calibration	Conversion	All slices	PE studies	PE-negative studies
Expanded (41 patients)	Width 700 / level 100 (submitted)	0.688	0.723	0.650

Table S2. Characteristics of the external images flagged by the gate (Reviewer 2, Question 1)

(a) Rules involved (RSNA-STR, submitted conversion; 288 of 1824 slices flagged; a slice can meet more than one rule). 47 of 76 studies had at least one flagged slice, 5 had more than half flagged and none had all slices flagged; the five studies with the most flags contributed 89 of the 288 flags.

Rule	Flagged slices
Low histogram correlation	183
Low entropy	68
Histogram distance	53
High extreme-value fraction	47
Low Laplacian variance	37
Low edge density	1

(b) Passed versus flagged slices (medians) and the study-level Spearman correlation of each characteristic with the study flag rate.

Characteristic	Passed	Flagged	Study-level ρ with flag rate
Body fraction of the image	0.563	0.635	0.35
Image-noise estimate	1.017	1.599	-0.03
Mean gray level	41.918	54.189	0.35
Pixel spacing (mm)	0.684	0.670	-0.26

Characteristic	Passed	Flagged	Study-level ρ with flag rate
Normalized slice position (0 = inferior)	0.565	0.174	–

(c) PE-positive images. 204 of 218 slices with PE marked on the image (0.936) passed the gate.

(d) Pass rate by manufacturer and reconstruction (LIDC-IDRI non-contrast CT, institutional conversion; manufacturer tags are removed in the RSNA-STR subset).

Manufacturer	Kernel	Slice thickness (mm)	Series	Pass rate
Ge	LUNG	2.5	1	1.000
Ge	STANDARD	2.5	7	0.908
Siemens	B30f	1.8	1	0.697
Siemens	B30f	1.8	1	1.000
Siemens	B30f	1.8	1	1.000
Siemens	B31f	3	3	0.306
Siemens	B45f	3	2	0.657
Toshiba	FC01	3	8	0.832
**Ge (all)**			8	**0.920**
**Siemens (all)**			8	**0.660**
**Toshiba (all)**			8	**0.832**

Table S3. Sensitivity to the Gaussian width σ (Reviewer 1, Comment 3)

Generator and refiner fixed; σ was not re-selected. Final output, patient medians.

(a) Distribution/contrast and input-structure metrics

Cohort	σ (px)	Mediastinal EMD ↓	Support EMD ↓	Gap error ↓	EPI ↑	Novel-edge rate ↓	Lost-edge rate ↓
Test (30 patients)	0.5	7.22	15.28	4.50	0.696	0.0155	0.0058
	0.75	7.17	15.34	4.50	0.749	0.0129	0.0056
	1 (used)	7.19	15.38	4.25	0.779	0.0111	0.0053
	1.5	7.23	15.45	4.25	0.811	0.0092	0.0046
	2	7.26	15.53	4.00	0.822	0.0081	0.0040
Validation (22 patients)	0.5	6.08	14.52	5.50	0.697	0.0144	0.0038
	0.75	6.08	14.58	5.25	0.748	0.0127	0.0042
	1 (used)	6.08	14.62	5.50	0.777	0.0107	0.0041
	1.5	6.06	14.70	5.25	0.810	0.0089	0.0030
	2	6.05	14.80	5.25	0.824	0.0081	0.0027

(b) Correlation between output and input in band-pass components (difference of Gaussians, test cohort).

Method	0.5–1 px	1–2 px	2–4 px	4–8 px	8–16 px
Stage 2 (generator) alone	0.954	0.984	0.972	0.955	0.940
Stage 2 + frequency split	0.995	0.990	0.974	0.957	0.941
Final output	0.989	0.986	0.973	0.958	0.944
CUT	0.734	0.934	0.954	0.956	0.952
CLAHE	0.935	0.964	0.977	0.981	0.984

(c) Where the change is placed (test cohort, 3,428 slices). Change = output − input; ratio = mean change in

the 2-pixel ring outside the vascular support divided by the mean change inside it.

σ (px)	Ring-to-support ratio	Mean change inside support (gray levels)	Mean |change| > 4 px from support
0.5	0.644	22.65	6.83
0.75	0.646	22.60	6.75
1 (used)	0.647	22.56	6.72
1.5	0.651	22.48	6.67
2	0.655	22.39	6.64
Registered CTPA of the same study	0.111	40.15	24.66

Table S4. Patient-level paired effects on the test cohort (30 patients; Reviewer 1, Comments 4 and 6)

Change = final output − comparator. Counts: patients with a positive / zero / negative change. Holm correction over the eight metrics of each comparison.

Comparison	Metric	Comparator	Final output	Median change (95% CI)	+ / 0 / −	Holm p
vs non-contrast input	Mediastinal EMD ↓	14.9217	7.1889	-8.8151 (-13.3230 to -5.0895)	4/0/26	8.9×10^{-5}
vs non-contrast input	Body EMD ↓	12.0368	4.8378	-5.9781 (-8.6755 to -4.6644)	2/0/28	4.1×10^{-7}
vs non-contrast input	Support EMD ↓	42.1413	15.3783	-21.8433 (-25.7605 to -20.5831)	1/0/29	1.5×10^{-8}
vs non-contrast input	Gap error ↓	11.0000	4.2500	-6.5000 (-9.0000 to -4.7500)	1/0/29	3.1×10^{-7}
vs non-contrast input	EPI ↑	1.0000	0.7788	-0.2212 (-0.2326 to -0.2086)	0/0/30	1.5×10^{-8}
vs non-contrast input	Sharpness ratio	1.0000	1.0789	0.0789 (0.0752 to 0.0865)	30/0/0	1.5×10^{-8}
vs non-contrast input	Novel-edge rate ↓	0.0000	0.0111	0.0111 (0.0086 to 0.0161)	30/0/0	1.5×10^{-8}
vs non-contrast input	Lost-edge rate ↓	0.0000	0.0053	0.0053 (0.0037 to 0.0076)	30/0/0	1.5×10^{-8}
vs CLAHE	Mediastinal EMD	13.6055	7.1889	-5.7468 (-6.5907 to -	3/0/27	2.3×10−

Comparison	Metric	Comparator	Final output	Median change (95% CI)	+ / 0 / −	Holm p
	↓			3.8066)		6
vs CLAHE	Body EMD ↓	10.5073	4.8378	-4.5954 (-6.4301 to -2.9117)	4/0/26	9.4×10−6
vs CLAHE	Support EMD ↓	23.7247	15.3783	-6.9517 (-9.5674 to -5.4111)	1/0/29	1.3×10−6
vs CLAHE	Gap error ↓	17.2500	4.2500	-12.0000 (-15.5000 to -9.5000)	1/1/28	9.4×10−6
vs CLAHE	EPI ↑	0.7775	0.7788	0.0079 (0.0005 to 0.0164)	21/0/9	0.070
vs CLAHE	Sharpness ratio	1.7624	1.0789	-0.6809 (-0.8207 to -0.5560)	0/0/30	1.5×10−8
vs CLAHE	Novel-edge rate ↓	0.0254	0.0111	-0.0139 (-0.0209 to -0.0097)	0/0/30	1.5×10−8
vs CLAHE	Lost-edge rate ↓	0.0004	0.0053	0.0049 (0.0032 to 0.0071)	30/0/0	1.5×10−8
vs CUT	Mediastinal EMD ↓	9.9117	7.1889	-2.4040 (-4.2866 to 0.6660)	11/0/19	0.154
vs CUT	Body EMD ↓	6.1071	4.8378	-0.2613 (-1.3699 to 0.3376)	13/0/17	0.289
vs CUT	Support EMD ↓	11.8999	15.3783	1.2694 (-0.0998 to 5.8495)	19/0/11	0.154
vs CUT	Gap error ↓	7.2500	4.2500	-1.0000 (-3.2500 to 0.2500)	10/2/18	0.166
vs CUT	EPI ↑	0.3896	0.7788	0.3832 (0.3781 to	30/0/0	1.5×10−

Comparison	Metric	Comparator	Final output	Median change (95% CI)	+ / 0 / −	Holm p
				0.3949)		8
vs CUT	Sharpness ratio	1.1737	1.0789	-0.0904 (-0.1597 to -0.0005)	10/0/20	0.073
vs CUT	Novel-edge rate ↓	0.0201	0.0111	-0.0089 (-0.0137 to -0.0068)	0/0/30	1.5×10−8
vs CUT	Lost-edge rate ↓	0.0429	0.0053	-0.0354 (-0.0432 to -0.0293)	0/0/30	1.5×10−8
stage3 vs cyclegan_qcpass	Mediastinal EMD ↓	6.9913	7.1889	0.4903 (-0.0845 to 0.8752)	19/0/11	0.133
stage3 vs cyclegan_qcpass	Body EMD ↓	5.1245	4.8378	-0.0059 (-0.3139 to 0.1387)	15/0/15	0.612
stage3 vs cyclegan_qcpass	Support EMD ↓	18.6884	15.3783	-1.8010 (-2.6305 to -1.5773)	1/0/29	1.5×10−6
stage3 vs cyclegan_qcpass	Gap error ↓	5.0000	4.2500	-1.0000 (-1.2500 to 0.0000)	1/11/18	4.5×10−4
stage3 vs cyclegan_qcpass	EPI ↑	0.8020	0.7788	-0.0278 (-0.0327 to -0.0202)	0/0/30	1.5×10−8
stage3 vs cyclegan_qcpass	Sharpness ratio	1.0174	1.0789	0.0635 (0.0601 to 0.0667)	30/0/0	1.5×10−8
stage3 vs cyclegan_qcpass	Novel-edge rate ↓	0.0085	0.0111	0.0020 (0.0013 to 0.0029)	30/0/0	1.5×10−8
stage3 vs cyclegan_qcpass	Lost-edge rate ↓	0.0051	0.0053	0.0002 (0.0001 to 0.0002)	30/0/0	1.5×10−8

Table S5. Lesion versus intensity-matched control boxes (11 patients; Reviewer 1, Comment 5)

Contrast change relative to the non-contrast input (gray levels); difference = lesion change − control change within each patient.

Method	Lesion boxes	Control boxes	Difference (95% CI)	Wilcoxon p	Holm p (4 methods)	Positive
CLAHE	23.2	32.4	-3.68 (-7.25 to 1.66)	0.042	0.126	4/11
CUT	23.6	15.4	4.57 (1.45 to 8.19)	0.024	0.098	9/11
Stage 2 + frequency split	28.2	26.9	1.35 (-2.17 to 7.89)	0.240	0.350	7/11
Final output	28.2	26.9	1.34 (-0.62 to 5.92)	0.175	0.350	8/11

With alternative weighting of repeated studies (study-balanced; slice- and study-balanced), the median difference for the final output was 2.0–2.2 gray levels (Wilcoxon $p = 0.054$).

Table S6. Repeated training runs (Reviewer 1, Comment 6)

Both learned stages retrained with the submitted configuration; test cohort, final output, patient medians. The refiner validation criterion (EPI decrease ≤ 0.03 relative to the composition) was checked on the validation cohort before testing.

Run	Mediastinal EMD	Support EMD	Gap error	EPI	Novel-edge rate	Lost-edge rate	Refiner validation criterion
submitted	7.19	15.38	4.25	0.779	0.0111	0.0053	submitted run
seed 101	7.61	19.78	6.25	0.819	0.0078	0.0050	met
seed 202	7.29	15.54	3.50	0.780	0.0092	0.0183	not met
seed 303	7.46	13.52	2.75	0.792	0.0077	0.0200	not met
seed 404	7.46	16.93	4.12	0.817	0.0088	0.0049	met

Against CUT, every run had a higher EPI and lower novel- and lost-edge rates (all $p < 0.05$); CUT had a lower

support EMD in every run (median paired difference 0.57 to 4.00; significant in 2 of 4 runs).

Table S7. Change of the correction between adjacent slices on DICOM-ordered volumes (Reviewer 1, Comment 6)

74 non-contrast examinations (49 patients) ordered by DICOM image position; correction = output − input; mean absolute change between adjacent slices, patient medians (gray levels). The same measure on consecutive display images of the test cohort gave 5.01 (final output), 10.74 (CUT) and 8.28 (CLAHE).

Method	Within the body	Within the mediastinal ROI	Per mm of slice spacing
Stage 2 + frequency split	4.65	8.06	1.55
Final output (Stage 1+2+3)	5.43	9.18	1.79
CUT	11.44	15.69	3.75
CLAHE	8.96	10.67	2.91

Comparison (within the body)	Median paired difference (95% CI)	Final output lower	Wilcoxon p
Final output - CUT	-5.74 (-6.18 to -5.25)	49/49	3.6×10^{-15}
Final output - CLAHE	-3.24 (-3.89 to -2.73)	49/49	3.6×10^{-15}
Final output - Stage 2 + frequency split	0.74 (0.62 to 0.90)	0/49	3.6×10^{-15}

Table S8. External non-contrast CT: complete pipeline (Reviewer 1, Comment 2)

(a) Accepted series (LIDC-IDRI; gate pass rate with the institutional and the submitted conversion).

Series	Manufacturer	Model	Kernel	Slices	Thickness (mm)	Pass, institutional	Pass, submitted
LIDC-IDRI-0016	Ge	LightSpeed16	STANDARD	141	2.5	1.00	0.00
LIDC-IDRI-	Ge	LightSpeed16	STANDARD	141	2.5	0.89	0.16

Series	Manufacturer	Model	Kernel	Slices	Thickness (mm)	Pass, institutional	Pass, submitted
0028							
LIDC-IDRI-0063	Ge	LightSpeed16	STANDARD	133	2.5	1.00	0.59
LIDC-IDRI-0108	Ge	LightSpeed VCT	STANDARD	143	2.5	1.00	0.34
LIDC-IDRI-0177	Ge	LightSpeed16	STANDARD	133	2.5	0.92	0.02
LIDC-IDRI-0187	Ge	LightSpeed VCT	STANDARD	143	2.5	0.57	0.33
LIDC-IDRI-0190	Ge	LightSpeed Plus	STANDARD	127	2.5	1.00	0.99
LIDC-IDRI-0196	Ge	LightSpeed Plus	LUNG	133	2.5	1.00	0.84
LIDC-IDRI-0104	Toshiba	Aquilion	FC01	105	3	0.89	0.10
LIDC-IDRI-0346	Toshiba	Aquilion	FC01	105	3	0.75	0.17
LIDC-IDRI-0354	Toshiba	Aquilion	FC01	113	3	0.67	0.17
LIDC-IDRI-0368	Toshiba	Aquilion	FC01	111	3	1.00	0.11
LIDC-IDRI-0642	Toshiba	Aquilion	FC01	112	3	0.75	0.09

Series	Manufacturer	Model	Kernel	Slices	Thickness (mm)	Pass, institutional	Pass, submitted
LIDC-IDRI-0724	Toshiba	Aquilion	FC01	122	3	0.85	0.17
LIDC-IDRI-0945	Toshiba	Aquilion	FC01	124	3	1.00	0.02
LIDC-IDRI-1008	Toshiba	Aquilion	FC01	115	3	0.73	0.11
LIDC-IDRI-0103	Siemens	Sensation 16	B31f	111	3	0.08	0.00
LIDC-IDRI-0127	Siemens	Sensation 16	B31f	117	3	0.06	0.00
LIDC-IDRI-0294	Siemens	Sensation 16	B30f	175	1.8	0.70	0.00
LIDC-IDRI-0347	Siemens	Sensation 16	B31f	131	3	0.72	0.03
LIDC-IDRI-0349	Siemens	Sensation 16	B30f	179	1.8	1.00	0.97
LIDC-IDRI-0351	Siemens	Sensation 64	B45f	103	3	0.75	0.69
LIDC-IDRI-0381	Siemens	Sensation 64	B45f	101	3	0.56	0.01
LIDC-IDRI-0413	Siemens	Sensation 64	B30f	177	1.8	1.00	1.00

(b) Paired comparisons of the final output, 24 series (change = final output − comparator).

Comparison	Metric	Median paired change (95% CI)	Wilcoxon p
vs non-contrast input	Mediastinal EMD to external CTPA	-7.76 (-9.57 to -3.83)	2.1×10^{-4}

Comparison	Metric	Median paired change (95% CI)	Wilcoxon p
	↓		
vs non-contrast input	Body EMD to external CTPA ↓	-4.30 (-6.01 to -3.18)	1.1×10⁻4
vs non-contrast input	Gap error ↓	-7.75 (-9.50 to -6.00)	0.015
vs non-contrast input	EPI ↑	-0.2234 (-0.2439 to -0.2034)	1.2×10⁻7
vs non-contrast input	Novel-edge rate ↓	0.0134 (0.0056 to 0.0237)	1.2×10⁻7
vs non-contrast input	Lost-edge rate ↓	0.0036 (0.0018 to 0.0069)	1.2×10⁻7
vs CUT	Mediastinal EMD to external CTPA ↓	-3.11 (-7.11 to -1.73)	0.001
vs CUT	Body EMD to external CTPA ↓	1.59 (1.23 to 2.62)	0.101
vs CUT	Gap error ↓	0.50 (-6.00 to 3.00)	0.444
vs CUT	EPI ↑	0.4055 (0.3712 to 0.4387)	1.2×10⁻7
vs CUT	Novel-edge rate ↓	-0.0087 (-0.0198 to -0.0051)	3.9×10⁻6
vs CUT	Lost-edge rate ↓	-0.0285 (-0.0410 to -0.0213)	1.2×10⁻7

(c) The same comparisons for the 16 series analysed before the eight Toshiba series were added.

Comparison	Metric	Median paired change (95% CI)	Wilcoxon p
vs non-contrast input	Mediastinal EMD to external CTPA ↓	-5.85 (-8.83 to -1.62)	0.013
vs non-contrast input	Body EMD to external CTPA ↓	-3.46 (-6.01 to -1.14)	0.006
vs non-contrast input	Gap error ↓	-9.00 (-12.00 to -5.50)	0.026
vs non-contrast input	EPI ↑	-0.2101 (-0.2405 to -0.1733)	3.1×10⁻5
vs non-contrast input	Novel-edge rate ↓	0.0101 (0.0036 to 0.0189)	3.1×10⁻5

Comparison	Metric	Median paired change (95% CI)	Wilcoxon p
vs non-contrast input	Lost-edge rate ↓	0.0025 (0.0014 to 0.0064)	3.1×10^{-5}
vs CUT	Mediastinal EMD to external CTPA ↓	-2.44 (-5.03 to 1.15)	0.093
vs CUT	Body EMD to external CTPA ↓	1.55 (0.55 to 1.92)	0.159
vs CUT	Gap error ↓	1.00 (-6.00 to 4.00)	0.887
vs CUT	EPI ↑	0.4147 (0.3644 to 0.4449)	3.1×10^{-5}
vs CUT	Novel-edge rate ↓	-0.0075 (-0.0144 to -0.0023)	0.001
vs CUT	Lost-edge rate ↓	-0.0250 (-0.0410 to -0.0144)	3.1×10^{-5}

(d) By manufacturer (patient medians).

Manufacturer	Mediastinal EMD, input → final	Gap error, input → final	EPI, final / CUT	Lost-edge rate, final / CUT
Ge	16.50 → 9.82	15.0 → 9.0	0.777 / 0.374	0.0045 / 0.0368
Siemens	12.38 → 9.58	16.0 → 5.5	0.833 / 0.383	0.0013 / 0.0247
Toshiba	17.18 → 5.98	9.5 → 2.8	0.756 / 0.349	0.0057 / 0.0381

(e) Independent PE model on the full external volumes (PE status not annotated): predicted PE volume 11.9 mL on the input and 4.4 mL after enhancement (decrease in 19 of 24 patients; Wilcoxon $p = 1.4\times10^{-4}$); original DICOM 11.9 mL.

Table S9. Independent 3D PE model trained on CTPA (Reviewer 1, Comment 6; Reviewer 2, Question 3)

(a) Paired per-patient comparisons with the non-contrast input (false-positive volume in 25 normal patients; lesion-box sensitivity in 11 PE patients; Holm correction within each end point).

End point	Representation	Input	Representation	Patients with a decrease	Holm p
Predicted PE volume in normal patients (mL)	CLAHE	11.96	12.28	13/25	0.853
Predicted PE volume in normal patients (mL)	CUT	11.96	4.81	19/25	0.003
Predicted PE volume in normal patients (mL)	Stage 2 + frequency split	11.96	1.73	23/25	1.5×10^{-6}
Predicted PE volume in normal patients (mL)	Final output (Stage 1+2+3)	11.96	1.75	23/25	4.5×10^{-6}
Predicted PE volume in normal patients (mL)	Non-contrast CT, original DICOM	11.96	10.81	17/25	0.018
Lesion-box sensitivity	CLAHE	0.58	0.62	1/11	0.128
Lesion-box sensitivity	CUT	0.58	0.66	3/11	0.866
Lesion-box sensitivity	Stage 2 + frequency split	0.58	0.15	9/11	0.038
Lesion-box sensitivity	Final output (Stage 1+2+3)	0.58	0.15	8/11	0.043
Lesion-box sensitivity	Non-contrast CT, original DICOM	0.58	0.54	4/11	0.276

(b) Full operating range (stored probability maps re-thresholded at 254 levels; false-positive burden = median predicted volume per normal examination; sensitivity pooled over 248 lesion boxes; 95% patient-cluster bootstrap CI).

Representation	Lowest reachable false-positive volume (mL)	Sensitivity at 10 mL per normal examination	AUROC across thresholds
Non-contrast CT, original DICOM	6.55	0.569 (0.391 to 0.694)	0.312–0.441
Non-contrast display	7.52	0.581 (0.405 to 0.723)	0.335–0.398

Representation	Lowest reachable false-positive volume (mL)	Sensitivity at 10 mL per normal examination	AUROC across thresholds
(input)			
CLAHE	7.79	0.609 (0.439 to 0.774)	0.363–0.446
CUT	2.94	0.605 (0.419 to 0.751)	0.431–0.530
Stage 2 + frequency split	0.63	0.109 (0.048 to 0.201)	0.361–0.516
Final output (Stage 1+2+3)	0.75	0.125 (0.052 to 0.255)	0.370–0.491

(c) Study-level AUROC with alternative study scores.

Representation	Predicted volume (primary)	Maximum probability	Largest component	Components ≥ 100 mm^3
Non-contrast CT, original DICOM	0.418	0.472	0.429	0.425
Non-contrast display (input)	0.392	0.500	0.432	0.484
CLAHE	0.434	0.500	0.422	0.548
CUT	0.521	0.516	0.547	0.561
Stage 2 + frequency split	0.491	0.477	0.517	0.454
Final output (Stage 1+2+3)	0.470	0.448	0.495	0.469
Real CTPA, original DICOM	0.887	0.902	0.892	0.902
Real CTPA, display	0.854	0.865	0.859	0.896

(d) Replication in 28 further PE examinations (16 patients not used to train Stages 2–3, including the sealed-test patients; same 32 normal examinations; no lesion annotations).

PE cohort	Representation	AUROC (95% CI)
All 28 examinations	Real CTPA, original DICOM	0.785 (0.624 to 0.938)
	Non-contrast CT, original DICOM	0.521 (0.308 to 0.712)
	Non-contrast display (input)	0.533 (0.325 to 0.726)
	CUT	0.532 (0.355 to 0.738)
	CLAHE	0.619 (0.408 to 0.820)
	Final output (Stage 1+2+3)	0.474 (0.304 to 0.680)
Earliest examination per patient (16)	Real CTPA, original DICOM	0.842 (0.665 to 0.998)
	Non-contrast CT, original DICOM	0.434 (0.248 to 0.642)
	Non-contrast display (input)	0.463 (0.275 to 0.670)
	CUT	0.588 (0.399 to 0.794)
	CLAHE	0.611 (0.403 to 0.823)
	Final output (Stage 1+2+3)	0.506 (0.318 to 0.712)

Supplementary Figures

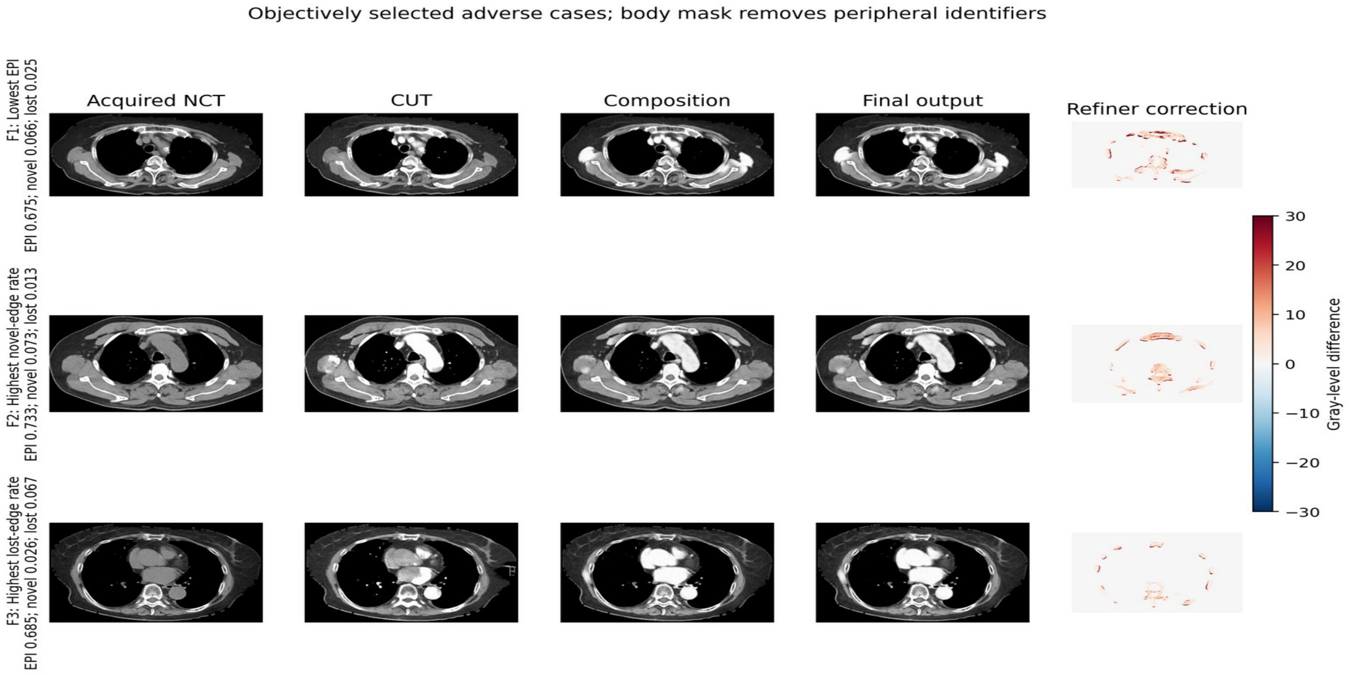

Figure S1. Objectively selected adverse slices of the test cohort: the slices with the lowest EPI, the highest novel-edge rate and the highest lost-edge rate among 3,428 slices, shown for the input, CUT, the composition and the final output, with the correction applied by the refinement (Reviewer 1, Comment 6).

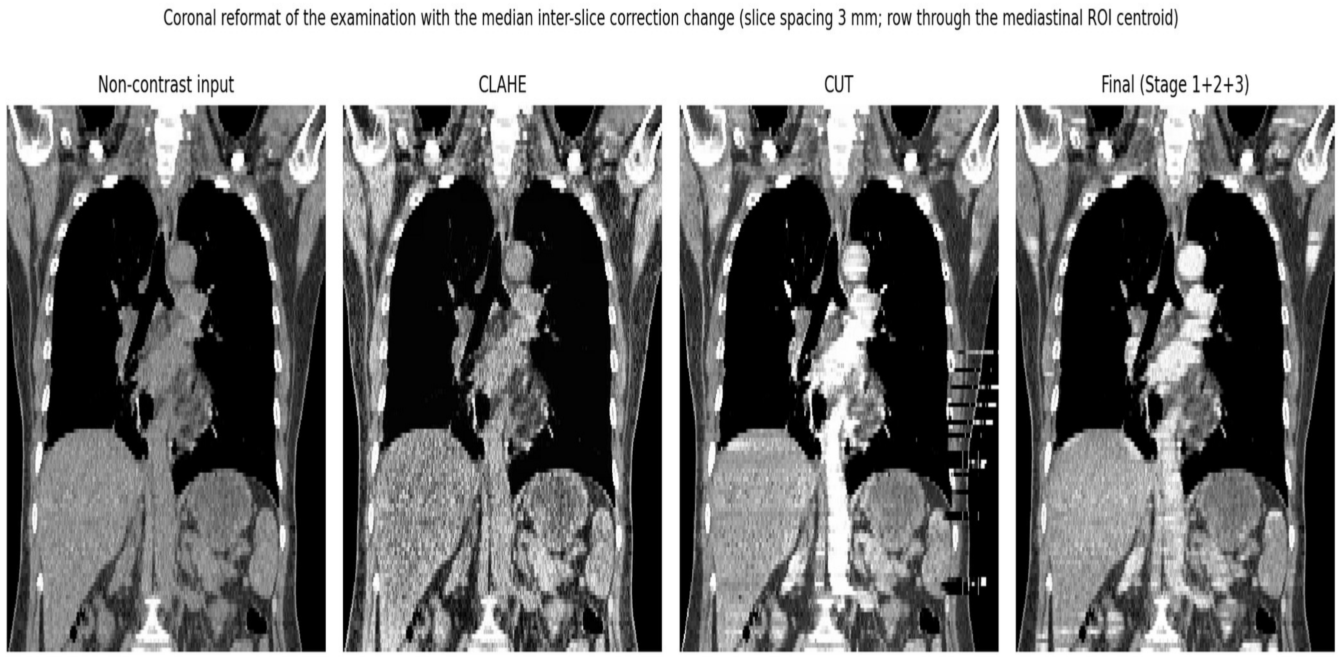

Figure S2. Coronal reformat of the non-contrast examination with the median inter-slice change of the correction (selection rule fixed before computation), for the input, CLAHE, CUT and the final output (Reviewer 1, Comment 6).
